# A Novel Adaptive Method for the Analysis of Next-Generation Sequencing Data to Detect Complex Trait Associations with Rare Variants Due to Gene Main Effects and Interactions

**DOI:** 10.1371/journal.pgen.1001156

**Published:** 2010-10-14

**Authors:** Dajiang J. Liu, Suzanne M. Leal

**Affiliations:** 1 Department of Molecular and Human Genetics, Baylor College of Medicine, Houston, Texas, United States of America; 2 Department of Statistics, Rice University, Houston, Texas, United States of America; Georgia Institute of Technology, United States of America

## Abstract

There is solid evidence that rare variants contribute to complex disease etiology. Next-generation sequencing technologies make it possible to uncover rare variants within candidate genes, exomes, and genomes. Working in a novel framework, the kernel-based adaptive cluster (KBAC) was developed to perform powerful gene/locus based rare variant association testing. The KBAC combines variant classification and association testing in a coherent framework. Covariates can also be incorporated in the analysis to control for potential confounders including age, sex, and population substructure. To evaluate the power of KBAC: 1) variant data was simulated using rigorous population genetic models for both Europeans and Africans, with parameters estimated from sequence data, and 2) phenotypes were generated using models motivated by complex diseases including breast cancer and Hirschsprung's disease. It is demonstrated that the KBAC has superior power compared to other rare variant analysis methods, such as the combined multivariate and collapsing and weight sum statistic. In the presence of variant misclassification and gene interaction, association testing using KBAC is particularly advantageous. The KBAC method was also applied to test for associations, using sequence data from the Dallas Heart Study, between energy metabolism traits and rare variants in *ANGPTL* 3,4,5 and 6 genes. A number of novel associations were identified, including the associations of high density lipoprotein and very low density lipoprotein with *ANGPTL4*. The KBAC method is implemented in a user-friendly R package.

## Author Summary

It has been demonstrated that both rare and common variants are involved in complex disease etiology. Until recently it was only possible to perform large scale analysis of common variants. With the development of next-generation sequencing technologies, detection and mapping of rare variants have been made possible. However, methods used to analyze common variants are not powerful for the analysis of rare variants. To address the problems of rare variant analysis working in a novel framework, the kernel-based adaptive cluster (KBAC) method was developed to perform gene/locus based analysis. The KBAC combines variant classification and association testing in a coherent framework. Through simulations motivated by population genetic and disease data, it is demonstrated that the KBAC has superior power to other rare variant analysis methods, especially in the presence of variant misclassification and gene interaction. Using data from the Dallas Heart Study, the KBAC method was applied to test for associations between energy metabolism traits and rare variants in *ANGPTL* 3,4,5 and 6 genes. A number of novel associations were identified. The KBAC method is implemented in a user-friendly R package.

## Introduction

Currently there is great interest in investigating the etiology of complex disease due to rare variants [1]–[6]. Until recently, indirect mapping of common variants has been the emphasis of complex trait association studies. It has been demonstrated that common variants tend to have modest phenotypic effects while rare variants are likely to have stronger phenotypic effects [7], although not strong enough to cause familial aggregation [8]. For mapping complex diseases due to common variants, instead of genotyping functional variants, tagSNPs are genotyped which act as a proxy for the underlying causal variants. For rare variant association studies, indirect mapping is not an optimal approach due to low correlations (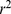) between tagSNPs and rare variants. Instead, direct mapping should be used, where functional variants are analyzed. In order to implement direct mapping, variants must first be identified. Large scale sequencing efforts have begun including the 1000 Genome Project, which will provide a better understanding of the allelic architecture of the genome and a detailed catalog of human variants. Next-generation sequencing technologies e.g. Roche 454, ABI SOLiD, and Illumina HiSeq, have made it feasible to carry-out rare variant association studies of candidate regions, exomes and genomes.

Gene interactions are believed to be involved in a broad spectrum of complex disease etiologies [9]. Although a number of methods have been developed to detect gene interactions between common variants [10]–[13], their detection has been limited [10]. There is evidence that rare variant interaction also plays a role in disease etiology. In direct association mapping of rare variants, one or more genetic loci are commonly jointly analyzed in order to aggregate information, for example genes with similar functions or residing in the same pathway [3], [4]. Therefore it is necessary to account for potential interactions between rare variants in different loci [14] and interactions between common and rare variants [15], [16].

Ideally, when carrying out direct mapping, only causal variants should be tested for associations. When DNA samples are sequenced, both causal and non-causal variants are uncovered. Bioinformatics tools [17], [18] or filters [1] can be used to predict functionality of variants, although tools such as PolyPhen [18] or SIFT [17]can have low sensitivity and specificity [6], [19]. Empirical studies have shown that predictive errors can be as high as 47% and 37% for PolyPhen and SIFT respectively [6]; therefore, their usefulness in selecting variants to be included in association analysis is limited. Even when functionality can be correctly inferred, whether the identified variants affect the phenotype of interest is still unknown. Two types of misclassifications of variant causality can frequently arise: 1.) non-causal variants are included in the analysis: a.) sequencing incorrectly identifies monomorphic sites as variant sites (false positive SNP discovery), b.) variants are falsely predicted to be functional or c.) variants are functional but non-causal; 2.) causal variants are excluded from the analysis: a.) due to locus heterogeneity, not all loci containing causal variants are included in the analysis, b.) region not sequenced, e.g. intronic variants, c.) variants not detected by sequencing assay (false negative SNP discovery) or d.) causal variants are falsely predicted to be non-functional.

Driven by the advancement of sequencing technologies and availability of data, statistical and computational methods are needed for analyzing sequence data. It has been demonstrated that methods used to analyze common variants are low powered when applied to the analysis of rare variants [20], [21]. Methods to analyze rare variants have been proposed [20], [21]; although they have clear advantages over implementing common variant analysis approaches, more powerful and robust methods need to be developed to analyze rare variant data, especially in the presence of variant misclassification or gene interactions.

The Kernel Based Adaptive Cluster (KBAC) was developed to overcome the problems of detecting rare variant associations in the presence of misclassification and gene interaction. Under the KBAC framework, data-based adaptive variant classification and testing of association are unified. The sample risk of a multi-site genotype is modeled using a mixture distribution with two components, where one component represents the distribution of sample risk of genotype if it is non-causal and the other component represents distribution of sample risks of causal genotypes. Ideally, if distributions for causal components were known, classification could first be performed and only the causal genotypes would be used in association studies. However, when searching for genotype-phenotype associations, it is usually unknown which variants are causal. Instead of performing an unrealistic two-step procedure, variant classification and association testing are unified in the KBAC framework. Continuous adaptive weighting which is implemented in the KBAC is preferable, particularly for low frequency alleles, than classifying variants and carrying out a stratified analysis, because increasing classification and shrinking size of strata can increase both type I and II error. For the KBAC, adaptive weighting procedure is implemented using the cumulative distribution functions for the multi-site genotype counts. Distributions of multi-site genotype counts are compared between cases and controls. Those multi-site genotypes that are enriched in cases will be up-weighted. Under the null hypothesis, the assigned weights asymptotically follow a uniform distribution. While under the alternative hypothesis, disease causal multi-site genotypes tend to be more frequent in cases than in controls. Therefore they are more likely to be adaptively up-weighted. The weighted multi-site genotype frequencies are aggregated and contrasted between cases and controls. In order to evaluate whether there is an association, significance of the KBAC can be assessed using either permutation or Monte Carlo approximation (See Methods and Figure S1).

The performance of the KBAC was compared to the weighted sum statistic (WSS) [21] the combined multivariate and collapsing (CMC) method [20], and the comparison of rare variants found exclusively in cases to those found only in controls (RVE) [3] using simulated data sets. Forward time simulation [22] assuming infinite-site Wright-Fisher model was used to generate population genetic data. Demographic change and purifying selection were both incorporated in the simulation, using parameters estimated from re-sequencing datasets from studies of African Americans (AA) and European Americans (EA) [23]. In addition to forward time simulation, population genetic data was also generated according to estimated site frequency spectrums (SFS) in AA and EA from the Dallas Heart Study (DHS) re-sequencing data of the *ANGPTL3*, *4*, *5*, and *6* genes.

For the simulated population data phenotypes were generated separately and motivated by epidemiological disease studies. Two types of main effects phenotypic model are considered: 1.) constant genetic effects for each causal variant and 2.) genetic effects inversely correlated with minor allele frequencies (MAF) of causal genetic variants. In order to evaluate the impact of variant misclassification, a variety of scenarios were examined where 1.) different proportions of non-causal variants were included in the analysis and 2.) different proportions of causal variants were excluded from the analysis.

Two disease models of gene interactions were also evaluated. The example of with-in gene interaction was motivated by Hirschsprung's disease [15], [16], where an interaction between a common polymorphism in the promoter region and multiple rare non-synonymous (NS) mutations in exonic regions of the *RET* gene is hypothesized [15], [16]. The example of between gene interaction is based on the observation that rare variants within the *CHEK2* gene increase risk of breast cancer in the absence of *BRCA1* and *BRCA2* mutations, but because of a shared pathway, the same *CHEK2* variants in the presence of high risk BRCA variants do not further increase risk [14], [24], [25].

Under each of the above scenarios, phenotype-genotype association testing is performed for rare NS variants. It is demonstrated that the KBAC has a clear advantage in power and robustness over other existing methods and this benefit is especially strong, when rare variant data is analyzed where there is either variant misclassification or gene interactions.

In order to further illustrate applications of the KBAC and other statistical methods, i.e., WSS, CMC, and RVE to carry-out association studies, energy metabolism traits and rare variants in *ANGPTL 3*, *4*, *5* and *6* genes obtained from sequence data were analyzed. In addition to identifying the originally reported association between triglyceride levels and *ANGPTL* 4, KBAC identified associations for a.) body mass index and *ANGPTL 5*, b.) diastolic blood pressure with *ANGPTL 6*, c.) high density lipoprotein with *ANGPTL 4*, d.) triglyceride levels with *ANGPTL 3* e.) very low density lipoprotein with *ANGPTL 3* and *ANGPTL 4*.

## Results

The results presented focus on simulations using simulated SFS from AA sequence data. Similar results are found for simulations using simulated SFS for EA and estimated SFS for AA and EA (Text S1 and Figures S2, S3, S4, S5, S6, S7, S8.). Although the power varies dependent on the underlying model used to generate the data, in all cases the KBAC is the most powerful method followed by the WSS, CMC and then the RVE.

### Rare Variant Frequency Distributions in Generated Case-Control Samples

Rare NS variants carrier information is summarized (Table 1) for replicates used in power comparisons in the presence of misclassifications. Under the phenotypic model with variable genetic effects, when all variants (both non-causal and causal variants) were analyzed, 5.5% of cases and 3.4% of controls are carriers, with carrier frequency in cases 61% higher than in controls. When only causal variants are included, the fractions of carriers in cases and in controls are 3.8% and 1.7% respectively. The case rare variant frequency is approximately 2.3 times of the controls frequency, which implicates that average ORs of uncovered rare variants lie between 2 to 3. For the phenotypic model with fixed genetic effects, the results are similar. The carrier frequency observed in cases is around 2.5 times the frequency in controls. Compared to the model with fixed effects, lower frequency rare causal variants have larger ORs for variable effects model. The probability that these low frequency rare variants are uncovered in a case-control sample is higher. Therefore, in all scenarios examined, more rare variants sites are uncovered for the model with variable effects. When all the variants are included, 11% more rare NS variants sites are uncovered for the model with variable effects. The number of rare variants sites that are exclusive to cases or controls is also higher under the variable effect model. For example, when 100% of the variant sites are included in the analysis, 47.4% and 41.1% of the sites are found exclusively in either cases or controls for the variable and the fixed effects model, respectively. For both models, within a single gene, very few cases and controls carry more than one rare variant.

**Table 1:** Rare variant summary statistics.

Scenario		Rare Variant Carrier Frequencies in Cases/Controls	Mean Number of Rare Variant Sites	Mean number of Rare Variant Sites Observed Exclusively in Cases/Controls	Proportions of Rare Variant Carriers with More than One Rare Variant in Case/Controls
**Phenotypic Model with Variable Genetic Effects Inversely Correlated with MAFs**					
Percentage of Causal Variants Excluded	20%	0.033/0.014	5.791	2.978	0.013/0.006
	40%	0.025/0.011	4.396	2.285	0.009/0.004
	60%	0.017/0.008	3.048	1.556	0.006/0.003
Percentage of Non-causal Variants Included	0%	0.038/0.017	6.942	3.609	0.016/0.006
	20%	0.041/0.02	7.614	3.859	0.018/0.008
	40%	0.044/0.023	8.501	4.274	0.019/0.009
	60%	0.048/0.027	9.535	4.645	0.021/0.012
	80%	0.051/0.03	10.539	5.044	0.022/0.014
	100%	0.055/0.034	11.665	5.53	0.025/0.016
**Phenotypic Model with Fixed Genetic Effects Unrelated to MAFs**					
Percentage of Causal Variants Excluded	20%	0.034/0.014	4.455	1.797	0.014/0.005
	40%	0.027/0.011	3.449	1.39	0.01/0.004
	60%	0.019/0.008	2.36	0.956	0.006/0.003
Percentage of Non-causal Variants Included	0%	0.041/0.017	5.325	2.158	0.017/0.007
	20%	0.043/0.019	5.996	2.439	0.018/0.008
	40%	0.047/0.023	7.058	2.875	0.02/0.01
	60%	0.05/0.027	8.007	3.259	0.022/0.013
	80%	0.054/0.03	8.931	3.565	0.024/0.013
	100%	0.057/0.034	10.047	4.132	0.026/0.015

The summary statistics are displayed for the generated replicates under main effects model with fixed and variable genetic effects using simulated SFS from AA population. Scenarios with different proportions of causal variants excluded and scenarios with different proportions of non-causal variants included were considered. The table displays for a given sample, the information on a) the average proportion of rare NS variant carriers among cases and controls; b) the mean number of rare NS variant sites; c) the mean number of rare NS variant sites that are exclusive to cases or controls; d) the average proportion of case and control rare NS variant carriers with more than one rare variant. For each scenario, a sample size of 1,000 cases and 1,000 controls were used. 2,000 replicates were generated for each scenario.

For the within gene interaction model (Table 2), similar patterns of NS variants sites and carrier frequencies are observed. When 100% of the rare variants are causal, 5.5% of the cases and 3.2% of the controls are carriers on average for a case-control sample. Due to interaction, frequency differences between cases and controls are mitigated. In the between gene interaction model (Table 2), higher case carrier frequency and more rare variants sites are observed for the high risk gene than for the low risk gene. The proportions of rare variants carriers for the two genes combined can be high, e.g. when 100% of the variants are causal, up to 12% of the cases can be rare variant carriers. Rare variants distributions can be found in the (Text S1) for main effects models (Table S1) and within and between gene interactions models (Table S2) using simulated SFS for EA, and for main effects models using estimated SFS for AA (Table S3) and EA (Table S4) with re-sequencing data from *ANGPTL3*, *4*, *5*, and *6* genes.

**Table 2:** Rare variant summary statistics.

Scenario			Rare Variant Carrier Frequencies in Cases/Controls	Mean Number of Rare Variant Sites	Mean number of Rare Variant Sites Observed Exclusively in Cases/Controls	Proportions of Rare Variant Carriers with More than One Rare Variant in Case/Controls
**Between Gene Interaction Model**						
Percentage of Causal Variants:	25%	Gene 1	0.049/0.035	7.348	3.612	0.022/0.015
		Gene 2	0.038/0.035	7.023	3.39	0.018/0.016
	50%	Gene 1	0.065/0.035	7.699	3.749	0.029/0.015
		Gene 2	0.042/0.034	7.174	3.475	0.019/0.016
	75%	Gene 1	0.079/0.034	8.146	4.024	0.035/0.015
		Gene 2	0.046/0.034	7.259	3.509	0.021/0.015
	100%	Gene 1	0.096/0.034	8.622	4.276	0.043/0.015
		Gene 2	0.049/0.035	7.432	3.553	0.023/0.016
**Within Gene Interaction Model**						
Percentage of Causal Variants	25%		0.037/0.032	9.109	2.999	0.016/0.014
	50%		0.043/0.032	9.295	3.026	0.02/0.014
	75%		0.048/0.031	9.352	3.003	0.022/0.014
	100%		0.055/0.032	9.627	3.042	0.028/0.014

The summary statistics are displayed for the generated replicates under within gene interaction model and between gene interaction model using simulated SFS from AA population. Scenarios with different proportions of causal variants were considered. The table displays for a given sample, the information on a) the average proportion of rare NS variant carriers among cases and controls; b) the mean number of rare NS variant sites; c) the mean number of rare NS variant sites that are exclusive to cases or controls; d) the average proportion of case and control rare NS variant carriers with more than one rare variant. For within gene interaction model, a sample size of 1,000 cases and 1,000 controls were used, and for the between gene interaction model, a sample size of 300 cases and 300 controls were used. 2,000 replicates were generated for each scenario.

### Evaluation of Type I Error

**Figure 1:**
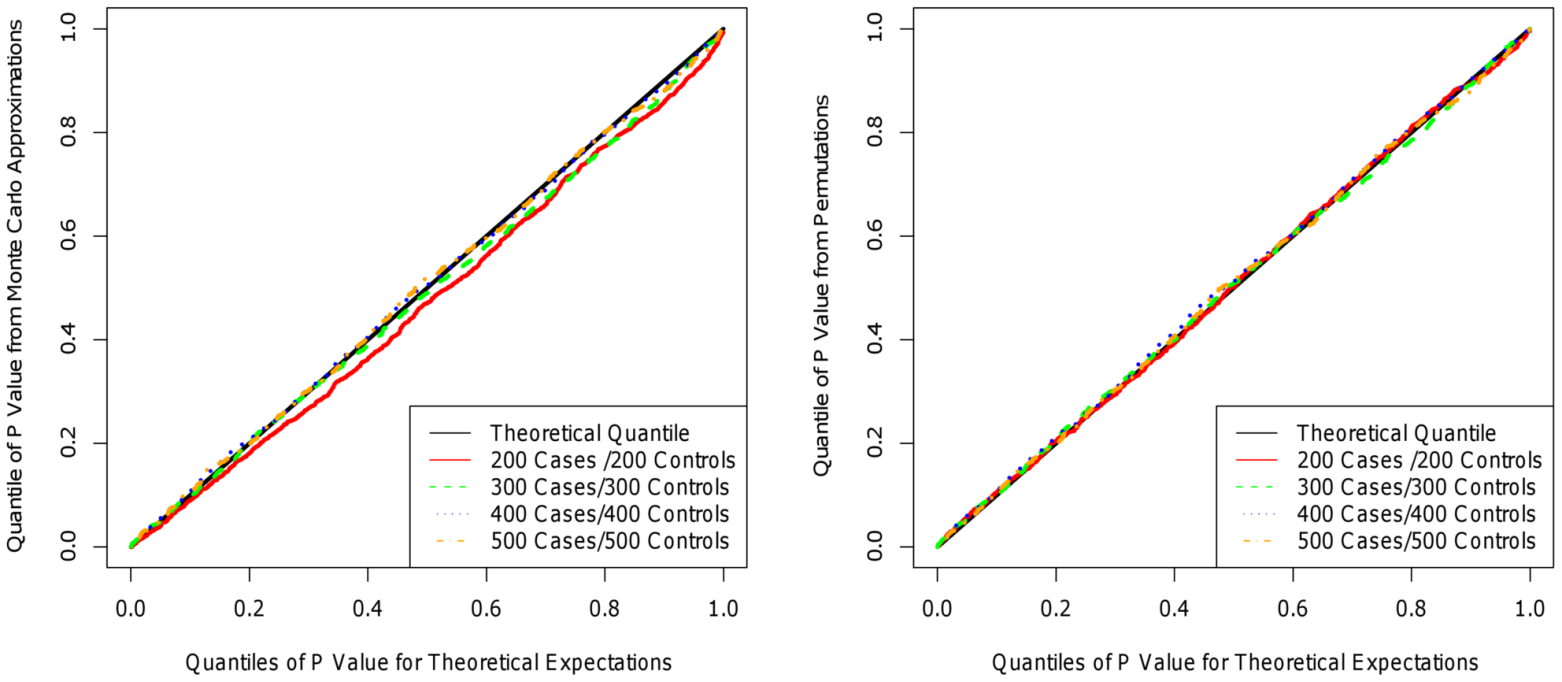
Quantile-Quantile (QQ) plot of p-values obtained from Monte Carlo approximation (left panel), permutation (right panel), and theoretical expectations. P-values were estimated using 10,000 iterations and 10,000 permutations for Monte Carlo approximation and permutation, respectively. Four sample sizes were investigated: 200 cases/200 controls; 300 cases/300 controls, 400 cases/400 controls, and 500 cases/500 controls. A total of 3,000 replicates were used to generate the QQ plot for each sample size.

When permutation was used to evaluate significance for the KBAC, type I error was well controlled, because p-values were obtained empirically. Additionally, in order to ensure that the type I error for RVE is well controlled permutation is also used to obtain empirical p-values. For the WSS [21], CMC [20] method, it was previously demonstrated that for the analysis of rare variants, their type I errors are well controlled [20]. For moderate sample sizes e.g. 400 cases/400 controls, the distributions of p-values for the Monte Carlo approximation are very close to those obtained using permutations and theoretical expectations (Figure 1) and additionally type I error is well controlled.

### Power Comparison

#### Main effects model without misclassification

**Figure 2:**
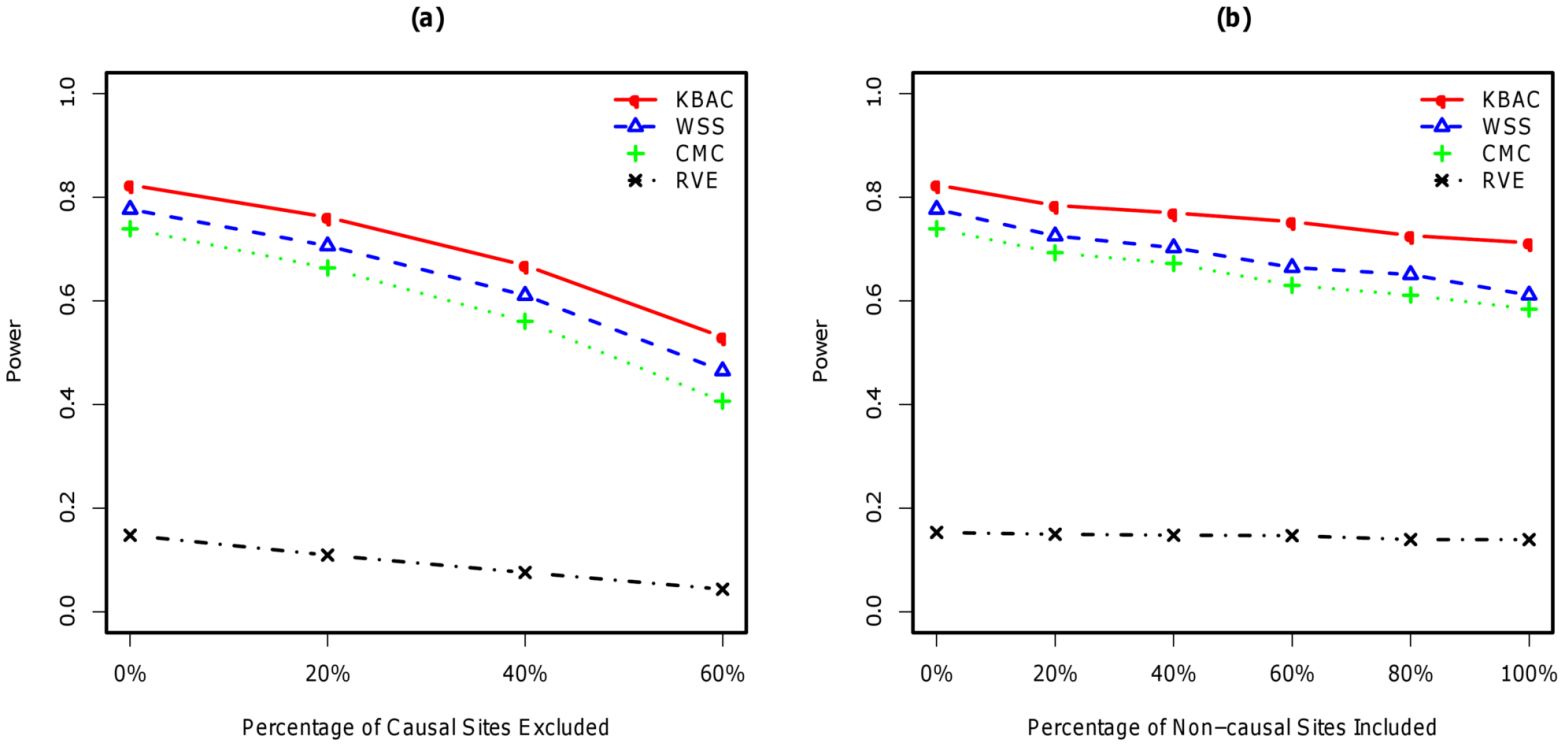
Impact of misclassifications under main effects model with fixed genetic effects using simulated SFS for AA. Each causal rare variant has an OR = 3.0. Power comparisons were made for the KBAC, WSS, CMC, and RVE when 0%∼60% of causal rare variants are excluded from the analysis (left panel) and when 0%∼100% of non-causal rare variants are included (right panel). A sample size of 1000 cases and 1000 controls was used for each scenario. P-values were empirically estimated using 5,000 permutations and power was evaluated for a significance level of 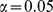 using 2,000 replicates for each scenario.

**Figure 3:**
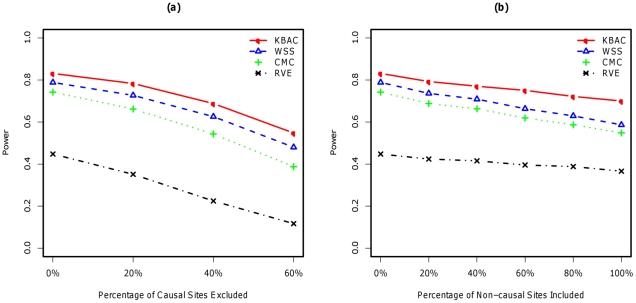
Impact of misclassifications under main effects model with variable genetic effects using simulated SFS for AA. The disease odds for causal variants are inversely correlated with their MAFs and within the range of 2∼20. Power comparisons were made for the KBAC, WSS, CMC, and RVE when 0%∼60% of causal rare variants are excluded from the analysis (left panel) and when 0%∼100% of non-causal rare variants are included (right panel). A sample size of 1000 cases and 1000 controls was used for each scenario. P-values were empirically estimated using 5,000 permutations and power was evaluated for a significance level of 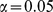 using 2,000 replicates for each scenario.

For main effects model with fixed genetic effects and no misclassification (Figure 2), the power 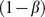 for KBAC, WSS CMC and RVE are respectively given by 82.5%, 77.7%, 73.9% and 14.8%. The power for RVE is much lower than the power for the other three methods. For the main effects model with variable genetic effects (Figure 3), the power for the four methods is given by 83.1%, 78.8%, 74.2% and 44.8%. The power of the RVE improves for the variable genetic effects model compared to the fixed genetics effect model; while the power for the other methods remains relatively unchanged. KBAC is consistently more powerful than WSS, CMC and RVE, e.g. for fixed effect model, KBAC is 6.1% more powerful than WSS, 11.6% more powerful than CMC, and 457.4% more powerful than RVE.

#### Impact of misclassification

Under both models (Figure 2, Figure 3), the power of all methods is negatively impacted by exclusions of causal variants and inclusions of non-causal variants at a varying degree. When non-causal variants are included in the analysis, KBAC is consistently more powerful and more robust than the other three methods. For example, when 100% of the non-causal variants are included, under the variable effects model, KBAC 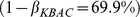 is 19.3% more powerful than WSS 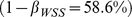, 27.6% more powerful than CMC 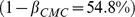, and 91.0% more powerful than RVE 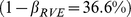. When compared under the fixed effects model, the advantage of KBAC 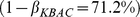 over WSS 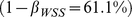, CMC 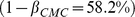 and RVE 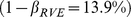 remains largely unchanged. For the scenarios where causal variants are missing, the relative performances of the methods remain to be in the order KBAC>WSS>CMC>RVE. For the variable effects model, the power advantage of WSS over CMC is greater than the advantage observed for the fixed effects model. For example, when 60% of the causal variants are excluded from the analysis, under the fixed effects model, the power for WSS drops 40.1% and the power of CMC drops 45.1%, while under the variable effects model, the power decreases for WSS and CMC are respectively 39.1%, 47.8%. The KBAC is more robust than the other methods: the power decreases under the fixed and variable effects models are respectively 34.1% and 35.6%, which are smaller than the decreases in power for WSS and CMC. Exclusion of causal variants from the analysis is more detrimental to power than inclusion of non-causal variants. Power comparisons with simulated SFS for EA can be found in (Figure S2) for fixed effects model and in (Figure S3) for variable effects model. Additionally, power comparisons with estimated SFS for AA are shown in (Figure S4) for fixed effects and in (Figure S5) variable effects models and that with estimated SFS for EA are displayed in (Figure S6) for fixed and in (Figure S7) for variable genetic effects models.

#### Within gene interaction model

**Figure 4:**
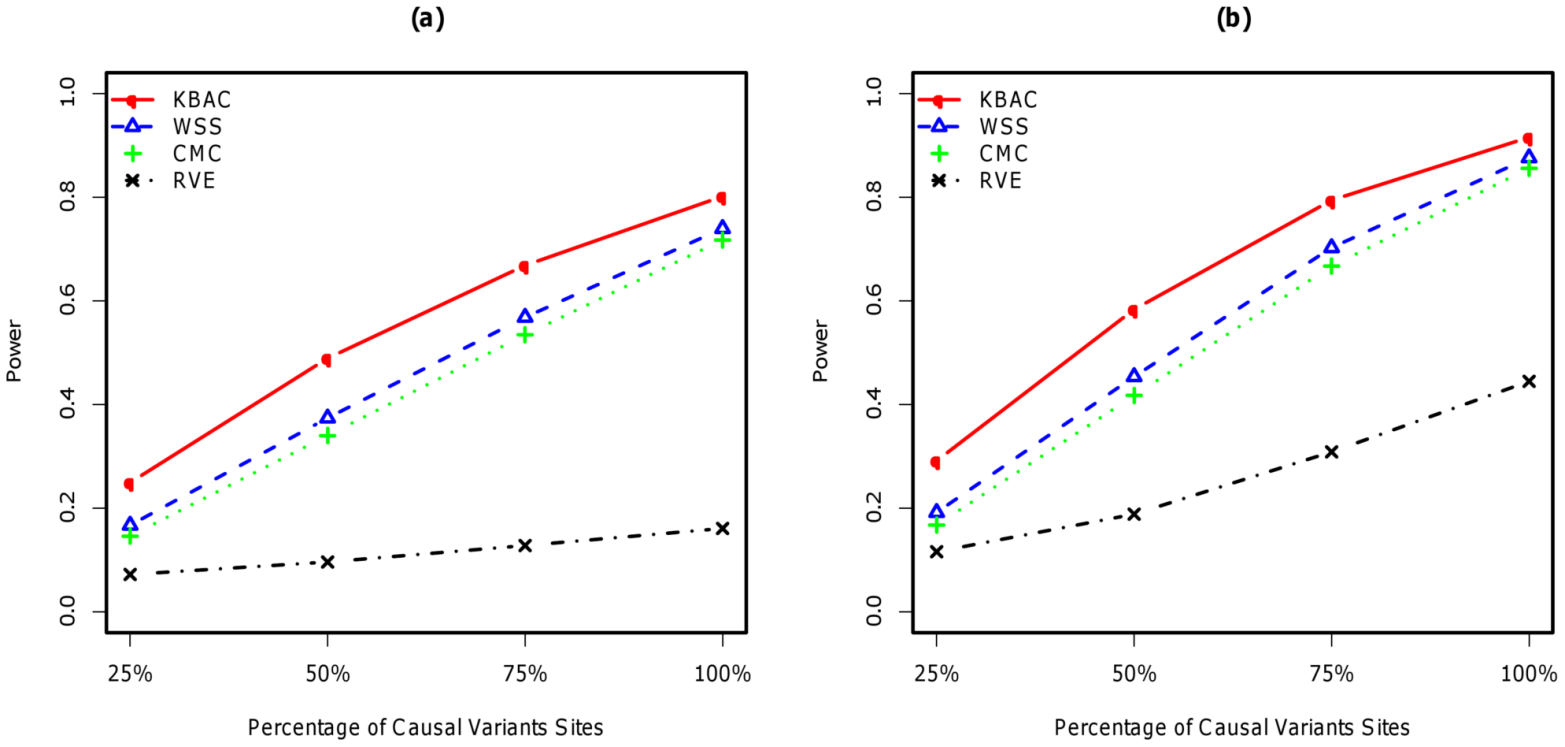
Power comparisons for within gene (left panel) and between gene interaction model (right panel) with simulated SFS for AA. Power was evaluated for the KBAC, WSS, CMC and RVE. A sample size of 1000 cases and 1000 controls were used for the within interaction model, and a sample size of 300 cases and 300 controls were used for the between gene interaction model. Scenarios with different proportions of causal variants were considered. P-values were empirically estimated using 5,000 permutations and power was evaluated for a significance level of 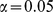 using 2,000 replicates.

Under the within gene interaction model, KBAC is consistently the most powerful method for all scenarios with different proportions of causal variants (Figure 4). The advantage of KBAC in the presence of interactions is apparent and its advantage over other methods becomes greater with increasing proportion of non-causal variants. For example, when all variants are causal, the power of KBAC is 8.4% higher than WSS, which is the second most powerful method. But when only 50% of all variants are causal, KBAC is 30.7% more powerful than WSS. RVE is the least powerful methods for all scenarios compared.

#### Between gene interaction model

In the between gene interaction model, power comparisons between the four methods remain similar (Figure 4). KBAC is consistently the most powerful method and is robust against inclusion of non-causal rare variant sites. Comparing the scenario where all variants are causal with the scenario where only 50% of the variants are causal, the power for KBAC drops 36.3%, while the power for WSS drops 48.2%. Power comparisons for within and between gene interaction models using simulated SFS based on sequence data from EA can be found in (Figure S8).

### *ANGPTL* Variants and Energy Metabolism in Humans

In order to further illustrate the application of KBAC and other rare variant analysis methods (i.e. WSS, CMC and RVE), rare variants in the *ANGTPL 3*,*4*,*5* and *6* genes were analyzed to determine whether they are associated with energy metabolism traits (Table 3). As in the original DHS study [26], the association of rare variants in the *ANGPTL3*,*4*,*5* and *6* genes with triglyceride (TG), low density lipoprotein (LDL), very low density lipoprotein (VLDL), high density lipoprotein (HDL), cholesterol, glucose, body mass index (BMI), systolic (SysBP) and diastolic blood pressure (DiasBP) were investigated. In the original DHS study, NS variants were analyzed using RVE, and significant associations were found between *ANGPTL3*, *ANGPTL 4* and TG as well as between *ANGPTL 6* and cholesterol [5], [6]. In this article NS variants, most of which are very rare [5], [6], were analyzed. Individuals with confounding factors (lipid lowering drugs, diabetes mellitus and heavy alcohol use) were removed for all analyses. Multiple associations were identified with KBAC but not with other approaches, i.e. the novel associations between *ANGPTL* 6 and DiaBP 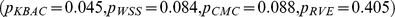, as well as between *ANGPTL* 3 and TG levels 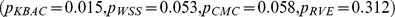. Additionally multiple novel associations were observed for analyses carried out with KBAC, WSS and CMC: 1.) *ANGPTL4* and VLDL 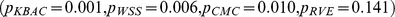; 2.) *ANGPTL5* and BMI 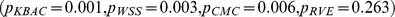; 3.) *ANGPTL4* and HDL 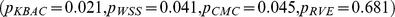 and 4.) the previously reported association between ANGPTL4 and TG levels 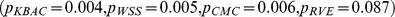. It should be noted that HDL and TG levels are negatively correlated (−0.42) and individuals with HDL levels in the lower quartile had an excess of rare variants in *ANGPTL4* compared to those individuals with HDL levels in the upper quartile, while those individuals with TG levels in the upper quartile had an excess of rare variants in *ANGPTL4* compared to those with TG levels in the lower quartile. The association detected by KBAC between ANGPTL4 and VLDL and between ANGPTL5 and BMI remains significant after correcting for multiple testing. RVE, on the other hand, detected associations between *ANGPTL 5*, *6* and glucose while the other three methods did not. We further investigated this association by applying a more stringent MAF cutoff 0.1% for the NS variants analyzed in *ANGPTL 5* and *6*. Using this new criterion both associations were detected by all methods (for *ANGPTL 5*, 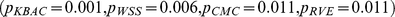 and for *ANGPTL 6*, 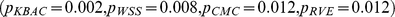).

**Table 3:** Association analyses of the *ANGPTL 3*,*4*,*5* and *6* gene variants with human energy metabolism phenotypes.

Phenotype	Gene Name	KBAC	WSS	CMC	RVE	Numbers of Carriers of Rare Variants Observed in Upper/Lower Quartiles	Number of Carriers of Rare Variants Observed Exclusively in either the Upper or Lower Quartiles
BMI	*ANGPTL3*	0.556	0.832	0.915	0.746	47/48	8/6
	*ANGPTL4*	0.999	0.331	0.412	0.104	62/71	2/7
	*ANGPTL5*	0.001*	0.003**	0.006**	0.263	83/51	5/1
	*ANGPTL6*	0.128	0.189	0.217	0.410	40/29	9/5
DiasBP	*ANGPTL3*	0.237	0.805	0.759	0.950	53/49	6/6
	*ANGPTL4*	0.784	0.437	0.445	0.086	56/63	3/9
	*ANGPTL5*	0.432	0.590	0.652	0.636	71/65	3/4
	*ANGPTL6*	0.045*	0.084	0.088	0.405	49/33	12/7
SysBP	*ANGPTL3*	0.455	0.965	1.000	0.919	49/48	7/6
	*ANGPTL4*	0.409	0.835	0.789	0.935	71/67	6/6
	*ANGPTL5*	0.106	0.498	0.602	0.053	77/71	10/2
	*ANGPTL6*	0.473	0.349	0.346	0.510	34/42	11/7
Cholesterol	*ANGPTL3*	0.950	0.299	0.326	0.906	40/49	7/7
	*ANGPTL4*	0.260	0.503	0.515	0.123	68/59	4/9
	*ANGPTL5*	0.353	0.697	0.783	0.778	68/63	8/7
	*ANGPTL6*	0.348	0.573	0.628	0.052	38/33	10/2
LDL	*ANGPTL3*	0.792	0.894	1.000	0.855	46/46	8/7
	*ANGPTL4*	0.508	0.695	0.709	0.064	66/60	4/11
	*ANGPTL5*	0.544	0.908	0.860	0.278	73/70	1/4
	*ANGPTL6*	0.307	0.745	0.813	0.388	39/36	9/5
HDL	*ANGPTL3*	0.834	0.992	1.000	0.237	50/51	2/7
	*ANGPTL4*	0.021*	0.041*	0.045*	0.681	84/62	7/6
	*ANGPTL5*	0.077	0.115	0.123	0.170	85/67	5/1
	*ANGPTL6*	0.143	0.211	0.239	0.513	43/33	6/9
TG	*ANGPTL3*	0.015*	0.053	0.058	0.312	34/52	6/11
	*ANGPTL4*	0.004**	0.005**	0.006**	0.087	46/76	2/8
	*ANGPTL5*	0.212	0.678	0.852	0.165	62/64	1/5
	*ANGPTL6*	0.683	0.664	0.709	0.057	35/32	15/6
VLDL	*ANGPTL3*	0.028*	0.047*	0.061	0.352	35/53	7/12
	*ANGPTL4*	0.001**	0.006**	0.010*	0.141	49/80	3/9
	*ANGPTL5*	0.265	0.941	1.000	0.263	67/68	1/5
	*ANGPTL6*	0.706	0.756	0.806	0.140	35/34	12/6
Glucose	*ANGPTL3*	0.485	0.589	0.612	0.690	49/55	5/7
	*ANGPTL4*	0.872	0.549	0.659	0.706	75/67	6/7
	*ANGPTL5*	0.407	0.896	0.862	0.021*	76/72	1/9
	*ANGPTL6*	0.196	0.198	0.239	0.026*	44/32	14/3

Nine phenotypes were analyzed: triglyceride (TG), high density lipoprotein (HDL), low density lipoprotein (LDL), very low density lipoprotein (VLDL), total cholesterol, glucose, body mass index (BMI), and systolic (SysBP) and diastolic (DiasBP) blood pressure. Analyses were carried-out including only NS variants. The KBAC, WSS, and CMC were used to analyze each trait and nominally significant p-values are indicated with an asterisk. The p values for KBAC, WSS and RVE were obtained empirically using 10,000 permutations, while the p-value for CMC was analytically calculated.

## Discussion

The KBAC method developed for association mapping of rare variants combines genotype classification and hypothesis testing in a coherent framework. The risk of each multi-site genotype is modeled as a mixture distribution with two components, among which only the component representing a non-causal genotype is known and is used in the adaptive weighting. Each multi-site genotype is continuously weighted using the non-causal component. The power of the KBAC as well as the other methods investigated can be affected by inclusion of non-causal mutations or exclusion of causal variants in the sample, to a varying degree. When non-causal variants are included in the analysis, the difference in rare variant carrier frequencies observed between cases and controls is mitigated. On the other hand, when causal variants are excluded from the association analysis, the marginal effect size of existing variants can vary considerably depending on whether missing causal variants exist on the same multi-site genotype. As a result, treating each variant (or multi-site genotype) interchangeably will incur loss of power, the severity of which will depend on the proportion of misclassified variants in the data. The performance of the KBAC is superior to the other approaches that were examined.

Bioinformatics tools [17], [18] and filters [1] can be used to determine which rare variants are potentially functional and should be included in the association analysis [1]. Their predictive accuracy, which can be low, is dependent on the amount of information available for the gene understudy. If bioinformatics tools are used to predict variant functionality and determine which variants should be included in the analysis it is best to loosen stringency, because the exclusion of causal variants is more detrimental to power than inclusion of non-causal variants. Whether or not bioinformatics tools are used as a screening tool, misclassification will occur therefore the robustness of KBAC to misclassification is particularly beneficial. Additionally in order to avoid potentially erroneous exclusion of causal variants due to locus heterogeneity, joint analysis of multiple putative genetic loci that carry similar functions or reside in the same pathway can be valuable.

It is of great interest to evaluate gene×gene interactions in the study of complex diseases. The KBAC analyzes multi-site genotypes (or multi-locus genotype), which can be beneficial in detecting gene interactions [11]. This property is especially important when multiple genetic loci are jointly analyzed in order to aggregate rare variants. Interactions are more likely to occur between genes involved in the same pathways. In addition, it has been hypothesized that functions of rare variants can be modulated by common variants [8]. Since the KBAC uses adaptive weighting instead of a fixed model, unknown patterns of gene interaction can be automatically integrated into the analysis. Through models motivated by Hirschsprung's disease and breast cancer, it is shown that in the presence of interactions the KBAC outperforms other approaches. An additional advantage of the KBAC is that kernel weights computed for adaptive weighting provide a measure with which the relative risk of each multi-site genotype can be assessed, for further replication studies.

The RVE method which compares the occurrence of variants which are exclusively observed in cases to those which are only observed in controls has the lowest power among all tests evaluated. The RVE method possesses undesired statistical properties by excluding those variants which are observed in both cases and controls. For all variants that are not fully penetrant, when sample size is large, they tend to appear in both case and control samples and would thus be excluded from the analysis using RVE. As a result, the RVE method is not asymptotically consistent; with increasing sample size power may be even lower than for smaller sample sizes [27].

Forward time simulations of locus genetic data incorporated both population demographic change and purifying selection. Both factors are known to impact SFS for observed rare variants (especially NS variants). Only NS variants were analyzed for comparing different methods, as it has been suggested that using NS variants will concentrate variations on functionally significant class of alleles, and increase signal to noise ratio [27]. There have been a number of studies on complex diseases which identified associations with NS variants [3], [5], [6]. When synonymous mutations are also considered in the analyses, higher proportions of non-causal variants may be introduced, so the adaptive property and the robustness of KBAC will be more advantageous.

Whether or not phenotypic effects of causal rare variants are inversely correlated with their MAF is unknown. Deleterious functional variants tend to have low frequencies [28], but the functional effect of a deleterious mutation may not be associated with the disease. On the other hand, for mutations involved in complex traits, they may not be at selective disadvantage due to the fact that most complex traits are late on-set and may not cause reductions in reproductive fitness. For both types of models, the advantage of KBAC is apparent. WSS and RVE perform better under the variable effects models, when only causal variants are present. This is because high risk causal variants are assigned higher weights. However, as low frequency non-causal variants also receive larger weights that negatively affect power, there are no measurable improvements of WSS compared to the model with fixed genetic effects. On the other hand, due to the adaptive nature of KBAC, the method performs consistently the best under both classes of models.

The KBAC test statistic does not have a closed form distribution; therefore it is necessary to evaluate significance either through permutation or using Monte Carlo approximation. For small sample sizes i.e. ∼≤400 cases and 400 controls, permutation is recommended, because it can be more reliable than Monte Carlo approximation. For larger sample sizes, Monte Carlo approximation not only controls type I error, but also the estimates of power do not differ from those obtained using permutations (data not shown). Permutation can be computationally intensive for large samples and/or genome-wide data where a large number of genetic regions are analyzed; therefore Monte Carlo approximation can be particularly advantageous to evaluate significance due to its computational efficiency.

A well known problem of genetic association studies is spurious findings due to population substructure and/or population admixture. For rare variant association analysis this problem can occur when study subjects are sampled from different populations and the distribution of non-causal variant sites and/or aggregate frequencies of non-causal variants differ between the sampled populations. To control for population stratifications, KBAC can be coupled with principal components analysis (PCA) [29] approach and eigenvector(s) can be included as covariates in the analysis (see Methods: Controlling for Confounders). PCA approach has been shown to be a powerful tool to accurately infer geographical locations [30], [31]. In addition, KBAC can also be used with clustering/matching based methods, such as structured association [32], [33] to control for population stratification.

The application of KBAC as well as WSS, CMC and RVE were further illustrated by the analyses of genes in *ANGPTL* family. In the analyses, all individuals with potentially confounding factors i.e. diabetics, alcoholics, and individuals treated with lipid lowering drug were excluded. In the original studies individuals were excluded based upon both their quantitative trait values and the confounding factors. For example, only individuals treated with lipids lowering drugs in the lower quartile of TGs were removed, but those in the upper quartile were included in the analysis. We believe excluding individuals based upon their quantitative trait values should not be done instead all individuals meeting the exclusion criteria should be removed from the analysis. KBAC performs consistently well, and identifies the most phenotype-genotype associations among all the approaches compared. The effects of mutant *ANGPTL* genes on lipoprotein lipase (LPL) have been studied through *in vitro* functional studies and *in vivo* mice studies. LPL has been known to affect glucose metabolism [34], cholesterol level [34]–[37], and blood pressure [38]. This biological evidence strengthens the support of the identified associations. Additionally, the association between variants in *ANGPTL*4 gene and triglyceride levels were successfully replicated using an independent dataset [5], [6].

Although the examples given are for the analysis of single regions and interaction between two regions, the KBAC can also be used to analyze entire exomes (or genomes). In order to control for family-wise error rate (FWER), it is sufficient to use a Bonferroni correction, since there will be little or no linkage disequilibrium between rare variants in different genes. It is thus not necessary to control the FWER using permutations. If exome sequencing is carried out and analysis is implemented gene by gene, given that human genome contains ∼20,000 genes, a significance level 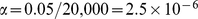 can be applied. The correction necessary for gene based association mapping of rare variants is less than the threshold currently used for genome-wide association studies [39] which is usually 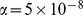.

The KBAC is a powerful tool to detect main association effects and gene interactions in large sequence data sets of candidate genes, exomes and in the future entire genomes. The KBAC is implemented in a user friendly R package and is available from the authors.

## Methods

### Sample Risk

Total sample size is denoted as 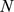, among which there are 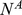 affected (A) and 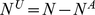 unaffected (U). It is assumed that there are 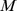 sites within the candidate region where rare variants are observed. The rare variant multi-site genotype for each “individual” is contained in a vector 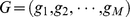, with the 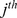 entry being the number of rare variants observed at 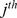 site, i.e. 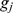 has value 2 if the site is homozygous for the rare allele, 1 if the site is heterozygous, 0 if the site is homozygous wild-type for the common major alleles. It is further assumed that 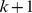 distinct multi-site genotype vectors, i.e 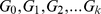 are observed, where 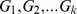 are multi-site genotypes with at least one rare variant and 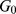 represents the wild-type genotype without any rare variants (i.e. a vector of all 0's). The sample risk for multi-site genotype 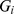 is defined as
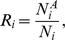
which is a consistent estimator of the ratio
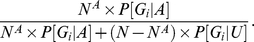



The ratio increases with disease penetrance of 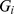 and provides a sample based measure of the relative risk.

The sample risk 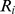 for multi-site genotype 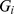 is modeled using a mixture distribution with two components, 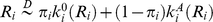. The component 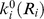 represents the distribution of the sample risk when multi-site genotype 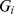 is non-causal and is known, while 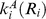 represents the unknown distribution of sample risk when 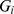 is causal. If the null hypothesis holds, all genotypes are non-causal, therefore, 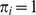. Under the alternative hypothesis, each genotype can be either causal or non-causal and the probabilities 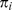 in the probabilistic mixtures are unknown.

If the mixture distribution under the alternative were known, then each genotype could be classified and only the causal genotypes would be used in the analysis. However, in disease gene mapping, the causality of variants is unknown. Instead of trying to ‘estimate’ 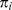 and 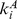 which are unknown, each multi-site genotype is adaptively weighted using only the known component, 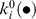. Each 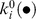 is called a kernel. The term kernel is borrowed from density estimation, where the density being estimated is spanned by a linear combination of kernel functions. The weight each rare genotype carries is given by the area under the curve which can be calculated as a generalized integral
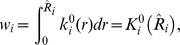
where 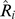 is the estimated sample risk for multi-site genotype 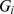.

Thereby, under the null hypothesis, the weights are uniformly distributed and under the alternative, greater weights can be placed on the multi-site genotypes that are enriched in cases. The genotypes with high sample risks will be given higher weights which can potentially separate causal from non-causal genotypes. Instead of classifying genotypes in a rigid manner with unknown likelihoods, this method weighs each genotype in a continuous fashion using only the known component 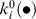 from the mixture density. The adaptive weighting procedure in the KBAC attains a good balance between classification accuracy and the number of parameters which are estimated.

### Choice of Kernels

Three types of kernels can be used to assign weights to each rare genotype; they are asymptotically equivalent. For small to moderate sample sizes, binomial and hyper-geometric likelihoods tend to work best, while for large sample sizes the asymptotic normal kernel is computationally efficient. All examples shown in this article were carried out using the hyper-geometric kernel.

#### Hyper-geometric kernel

Under the null hypothesis of no disease/gene associations, conditioning on the genotype counts 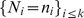 and the count of cases and controls 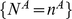, the number of diseased “individuals” having multi-site genotype 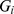 i.e. 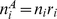 follows a hyper-geometric distribution with kernel function given by
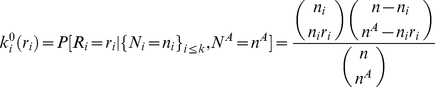
As this distribution is discrete, the integral is replaced by summations, i.e.
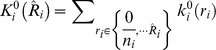



#### Marginal binomial kernel

Under the null hypothesis of no disease/gene association, conditioning on the genotype counts 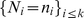, marginally, the number of disease “individuals” with genotype 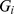, 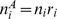 satisfies a binomial distribution, 
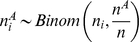
. Thus,
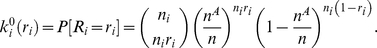



The weight as above is obtained through summations, i.e.
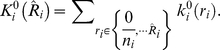



#### Asymptotic normal kernel

Under the null distribution, the sample risk for genotype 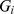 is asymptotically normal, i.e.
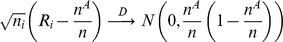
so the kernel is given by 
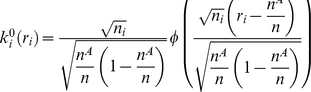
, where 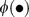 is the probability density function for a standard normal random variable. The weight for genotype 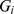 is given by the integral
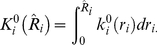



### Test Statistics

Each “individual” with multi-site genotype 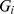 in the sample will be assigned weight 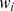. The weight is given by the kernel functions depending on the estimated sample risk 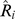 i.e. 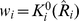. The weights assigned to rare genotypes are aggregated and contrasted between cases and controls.

The KBAC statistic is defined as 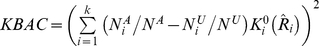, which compares the difference of weighted multi-site genotype frequencies between cases and controls. When a one sided alternative hypothesis is tested, e.g. the enrichment of causal variants in cases, a corresponding one sided version of KBAC can be used, i.e. 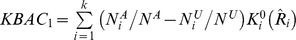. In this article, all power comparisons were based upon two sided tests for each method.

Standard permutation procedure is used to obtain empirical p-values for small sample sizes and for large sample sizes significance can be obtained through the Monte Carlo approximation. A graphical illustration of the KBAC statistic can be found in (Figure S9).

### Controlling for Confounders

In order to control for sample heterogeneities such as population stratification/admixture, it is desirable to be able to incorporate covariates in the association analysis. The kernel weights computed for the KBAC statistic can be used with logistic regression. For an individual 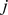 with multi-site genotype 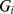, we define a variable for the kernel weight, i.e. 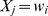. The logistic regression model for association testing has the form
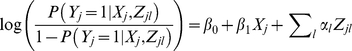
where 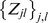 are the covariates such as age, sex or eigenvectors for genotypes.

A score statistic to test 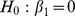 can be computed in closed form. Due to the complexities involved in computing kernel weights, the score statistic does not follow a normal distribution. Standard permutation procedure can be applied to evaluate the significance. When no additional covariates are controlled, the score function 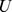 satisfies 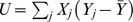 [40]. Simple algebraic manipulations will lead to the equivalence of the score function 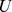 and the KBAC statistic (up to a constant scalar). In addition, when common variants in the gene are also hypothesized to play a role in the etiology of the phenotype of interest, their genotypes can be included as covariates and tested in a similar manner as for the CMC [20].

### Monte Carlo Approximation

#### Monte Carlo approximation under the null hypothesis

Although using permutation can provide an exact empirical distribution under the null hypothesis, it can be computationally prohibitive for large sample sizes and genome-wide association studies. A Monte Carlo method was developed which enables fast computation of p-values efficiently. Under the null hypothesis, conditioning on the genotype counts, 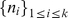 and the total number of cases and controls 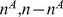, the number of cases 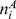 with multi-site genotype 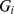 follows a binomial distribution 
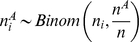
. Due to the low frequencies for each multi-site genotype containing rare variants, the 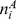
's are approximately independent of each other. Therefore, Monte Carlo simulation can be carried out as shown in algorithm 1:

Algorithm 1:

Step 1: Simulate a 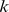-vector of independent binomials: 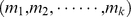, with 
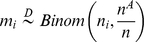


Step 2: Compute 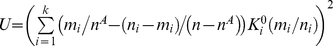


Step 3: Repeat step 1 and step 2 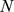 times and record each KBAC statistic calculated as 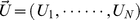. Through comparing the KBAC statistic calculated from the original data with the 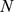 KBAC statistic from Monte Carlo simulation, the empirical p-value is given by 
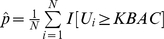
.

#### Monte Carlo approximation under the alternative hypothesis: power calculations

In this article power calculations were carried out empirically; haplotypes were generated using forward time simulations and case-control status was assigned via a linear log odds model. Power calculations can also be carried out using Monte Carlo approximation. Under the alternative hypothesis of disease-gene associations, it is assumed that the disease model is known (prevalence and population multi-site genotype frequencies 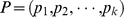 etc.) Therefore multi-site genotype frequencies for cases and controls can be assigned. The set of frequencies in cases and controls is denoted as 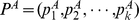, 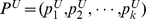. Conditioning on the genotype counts, 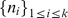 and the total number of cases and controls 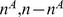, the number of cases 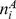 with the multi-site genotype 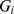 follows a binomial distribution, i.e.
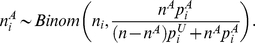



The power calculation under significance level 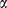 can be carried out in the following steps:

Algorithm 2:

Step 0: Generate 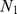 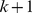-vectors 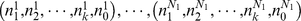 satisfying multinomial distribution i.e.
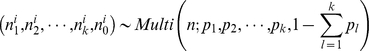



For each vector 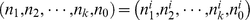, we follow step 1 to 4:

Step 1: Obtain an empirical distribution under the null by following step 1 and 2 in algorithm 1. The vector of 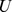
's obtained is denoted by 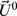 and the 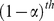 empirical quantile for 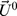 is denoted by 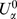


Step 2: Simulate a 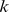-vector with independent binomials: 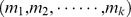, with 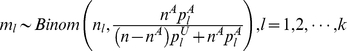


Step 3: Compute 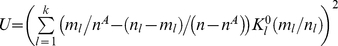


Step 4: Repeat step 2 and step 3 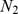 times and record each KBAC statistic calculated as 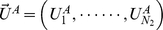. By comparing the KBAC statistic calculated from Monte Carlo simulation with 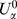, the empirical power conditional on 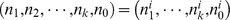 is given by 
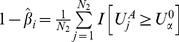
.

Step 5: The estimation of unconditional power is given by averaging 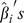, i.e. 
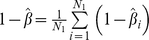


### Rare Variant Analysis Methods That Are Compared to the KBAC

The power of WSS, CMC and RVE were compared to KBAC in the article. A sketch of each method is provided here. More detailed descriptions can be found in the cited original reference. WSS was developed by Madsen and Browning [21]. It was designed to test for the differences of the number of mutations between cases and controls. Each mutation was weighted according to its frequency in controls, and lower frequency variants will be assigned higher weights. The statistical significance for the WSS statistic is obtained empirically through permutations.

CMC was developed by Li and Leal [20]. When applied to testing rare variant associations, multiple rare variants in the gene region are collapsed and carrier frequencies are compared between cases and control using Pearson's Chi-square test. The RVE [3], [4] was first introduced in the analysis of sequence data from Dallas Heart Study. It compares frequency of carriers of rare variants that are found exclusively in cases or controls using Fisher's exact test.

### Generation of Genetic Data

#### Simulation of demographic model and selections

To evaluate the performance of KBAC, population genetic data was generated using forward time simulation [22]. Genetic data from two populations, AA and EA were generated. The parameters for demographic changes and selection coefficients were estimated in Boyko et al [23]. For AA, a simple two-epoch model was used (Figure S10) while for EA, a six parameter complex bottleneck model was employed (Figure S11). Purifying selection was also simulated, with 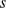 and 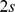 being the selective disadvantage of heterozygous and homozygous new mutations. Scaled fitness effect 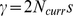 (where 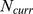 is the current effective population size) is assumed to follow a gamma distribution, which was shown to be parsimonious and fit the data well. Details of the choice of parameters can be found in (Text S1). A mutation rate of 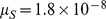 per nucleotide per generation is assumed. On average, the coding region for human gene is 1500 base pairs (bp) long [41], [42], therefore 1500 bps was used in the simulation to specify the locus scaled mutation rate. 100 haplotype pools were generated. When generating samples, one pool is randomly chosen for each replicate. The multi-site genotype of an “individual” is obtained by pairing two randomly sampled haplotypes.

#### Generation of genetic data using rare variant SFS

In order to further evaluate the performance of different methods, we generate genetic data using SFS estimated from genes in *ANGPTL* family (*ANGPTL 3*,*4*,*5* and *6*) from DHS. The SFS of rare variants was estimated using a method of moments approach (see Text S1 for details). When generating samples, estimated rare variants frequencies for one ANGPTL gene is randomly picked for each replicate. The multi-site genotype of an “individual” is generated according to the chosen set of gene variant frequencies.

#### Generation of phenotype data with only main effects

The disease status of each “individual” is assigned based upon their multi-site genotypes consisting of only those rare NS variants (MAF≤1%). Fifty-percent of the rare NS variant nucleotide sites were selected to be causal, where the rare mutant allele has an effect on the disease odds and the remaining rare variant sites are non-causal with no phenotypic effect. Two types of penetrance models were evaluated. In the first type of model, the genetic effects of causal variants are constant (OR = 3) regardless of their allele frequencies. For the second class of models, the genetic effects are inversely correlated with the MAFs. Disease odds of individual rare variants varies in the range of 2∼20. As a majority of rare variants are of extremely low frequencies, most of the uncovered rare variants in a case control sample have ORs between 2 and 4. This is compatible with surveys for multi-factorial diseases [8]. For both classes of penetrance specifications, a linear log odds model was applied to assign the affection status for each individual. Assignment of disease status continues until a sample of 1000 cases and 1000 controls is obtained for each replicate. To evaluate the effects of misclassification due to non-causal variants, scenarios were considered where 20%, 40%, 60%, 80%, and 100% of the non-causal variants with all of the causal variants were included in the sample. Additionally to evaluate the effect of misclassification due to exclusion of causal variants, 20%, 40%, and 60% of the causal variants were excluded from the analysis, while no non-causal variants are included in the analysis.

#### Generation of data with gene interactions

To evaluate the within gene interaction and between gene interaction models, 1000 cases/1000 controls and 300 cases/300 controls were generated for each replicate, respectively. For each model, 25% to 100% of the simulated rare variant sites are causal while the remaining rare variant sites are non-causal. For the within gene interaction model, one site with a common variant [MAF>20%] is randomly selected. The disease status of each “individual” is assigned based upon their multi-site genotype using a linear log odds model. The genetic effects of causal rare variants are modulated by the alleles at the chosen common variant site. Each causal rare variant increases disease risk with an OR of 3 only if the rare variant is on the same haplotype as the minor allele from the common variant site, otherwise the OR = 1. For the between gene interaction model, two unlinked genes are simulated for each “individual”. The disease status of each “individual” is assigned based upon their joint multi-site genotype at high risk gene 1 and low risk gene 2 using a linear log odds model. Each causal rare variant in gene 2 increases disease risk with an OR of 2.0 if there are no causal rare variants in gene 1; however, if there are rare causal variants in gene 1, the causal variants in gene 2 do not increase risk and each causal variant in gene 1 increases disease risk with an OR of 4.0 regardless of the genotype at gene 2. Mathematical illustrations of these two models are shown in Text S1.

### Analysis of Energy Metabolism Traits and Rare Variants in *ANGPTL 3*, *4*, *5* and *6*

The DHS dataset is a multi-ethnic population based probability sample [1830 AA, 601 Hispanics (H), 1045 EA, and 75 from other ethnicities] from Dallas County residents whose lipids and glucose metabolism have been characterized and recorded [26], [43]. In order to investigate how sequence variations in *ANGTPL3*, *4*, *5* and *6* influence energy metabolism in humans, coding regions of the four gene were sequenced using DNA samples obtained from 3551 participants in DHS [5]. A total of 348 nucleotide sites of sequence variations were uncovered in all four genes. Most of them are rare and 86% of them have MAFs below 1% [5]. Individuals with diabetes mellitus, heavy alcohol use, or who were taking lipids lowering drugs were removed from the all the analyses because these factors could be potential confounders. Additionally individuals who do not belong to the AA, H or EA ethnic groups were removed from the analysis. Following the original study [5], and to control for potential confounders [44] we stratified the sample by race, sex, and quantitative trait level. For each quantitative trait, to test if the rare variants are enriched in the expected extremes, individuals from bottom and top quartiles are used to mimic a case-control type of design. The KBAC, WSS, CMC and RVE were applied to carry-out the association analysis.

## Supporting Information

Figure S1 Schematic illustration of the permutation procedure used for evaluating statistical significance empirically.
(0.19 MB TIF)

Figure S2 Impact of misclassifications under main effects model with fixed genetic effects using simulated SFS for EA.
(0.18 MB TIF)

Figure S3 Impact of misclassifications under main effects model with variable genetic effects using simulated SFS for EA.
(0.18 MB TIF)

Figure S4 Impact of misclassifications under main effects model with fixed genetic effects using estimated SFS for AA from genes in *ANGPTL* family.
(0.18 MB TIF)

Figure S5 Impact of misclassifications under main effects model with variable genetic effects using estimated SFS for AA from genes in *ANGPTL* family.
(0.18 MB TIF)

Figure S6 Impact of misclassifications under main effects model with fixed genetic effects using estimated SFS for EA from genes in *ANGPTL* family.
(0.18 MB TIF)

Figure S7 Impact of misclassifications under main effects model with variable genetic effects using estimated SFS for EA from genes in *ANGPTL* family.
(0.18 MB TIF)

Figure S8 Power comparisons for within gene (left panel) and between gene interaction model (right panel) with simulated SFS for EA.
(0.19 MB TIF)

Figure S9 Graphical Illustrations for KBAC Statistic. In the KBAC framework, variants adaptive weighting and testing of associations are simultaneously performed. The statistical significance can be evaluated using either permutations or Monte Carlo approximations. For information on nomenclature used please refer to the Materials and Methods section.
(0.25 MB TIF)

Figure S10 Demographic History of AA with Two-Epoch Change.
(0.06 MB TIF)

Figure S11 Complex Demographic History of EA.
(0.09 MB TIF)

Table S1 Rare variant summary statistics. The summary statistics are displayed for the generated replicates under main effects model with fixed and variable genetic effects using simulated SFS from EA population. Scenarios with different proportions of causal variants excluded and scenarios with different proportions of non-causal variants included were considered. The table displays for a given sample, the information on a) the average proportion of rare NS variant carriers among cases and controls; b) the mean number of rare NS variant sites; c) the mean number of rare NS variant sites that are exclusive to cases or controls; d) the average proportion of case and control rare NS variant carriers with more than one rare variant. For each scenario, a sample size of 1,000 cases and 1,000 controls were used. 2,000 replicates were generated for each scenario.
(0.05 MB DOC)

Table S2 Rare variant summary statistics. The summary statistics are displayed for the generated replicates under within gene interaction model and between gene interaction model using simulated SFS from EA population. Scenarios with different proportions of causal variants were considered. The table displays for a given sample, the information on a) the average proportion of rare NS variant carriers among cases and controls; b) the mean number of rare NS variant sites; c) the mean number of rare NS variant sites that are exclusive to cases or controls; d) the average proportion of case and control rare NS variant carriers with more than one rare variant. For within gene interaction model, a sample size of 1,000 cases and 1,000 controls were used, and for between gene interaction model, a sample size of 300 cases and 300 controls were used. 2,000 replicates were generated for each scenario.
(0.04 MB DOC)

Table S3 Rare variant summary statistics. The summary statistics are displayed for the generated replicates under main effects model with fixed and variable genetic effects. Estimated SFS from AA population with *ANGPTL* dataset was used. Scenarios with different proportions of causal variants excluded and scenarios with different proportions of non-causal variants included were considered. The table displays for a given sample, the information on a) the average proportion of rare NS variant carriers among cases and controls; b) the mean number of rare NS variant sites; c) the mean number of rare NS variant sites that are exclusive to cases or controls; d) the average proportion of case and control rare NS variant carriers with more than one rare variant. For each scenario, a sample size of 1,000 cases and 1,000 controls were used. 2,000 replicates were generated for each scenario.
(0.04 MB DOC)

Table S4 Rare variant summary statistics. The summary statistics are displayed for the generated replicates under main effects model with fixed and variable genetic effects. Estimated SFS from EA population with *ANGPTL* dataset was used. Scenarios with different proportions of causal variants excluded and scenarios with different proportions of non-causal variants included were considered. The table displays for a given sample, the information on a) the average proportion of rare NS variant carriers among cases and controls; b) the mean number of rare NS variant sites; c) the mean number of rare NS variant sites that are exclusive to cases or controls; d) the average proportion of case and control rare NS variant carriers with more than one rare variant. For each scenario, a sample size of 1,000 cases and 1,000 controls were used. 2,000 replicates were generated for each scenario.
(0.04 MB DOC)

Text S1 Supplementary Material.
(0.25 MB DOC)
