# Supplementary figures and images for: A Novel Adaptive Method for the Analysis of Next-Generation Sequencing Data to Detect Complex Trait Associations with Rare Variants Due to Gene Main Effects and Interactions
Source: PLoS Genet. 2010 Oct 14;6(10):e1001156. doi: 10.1371/journal.pgen.1001156 (PMC2954824; doi:10.1371/journal.pgen.1001156)

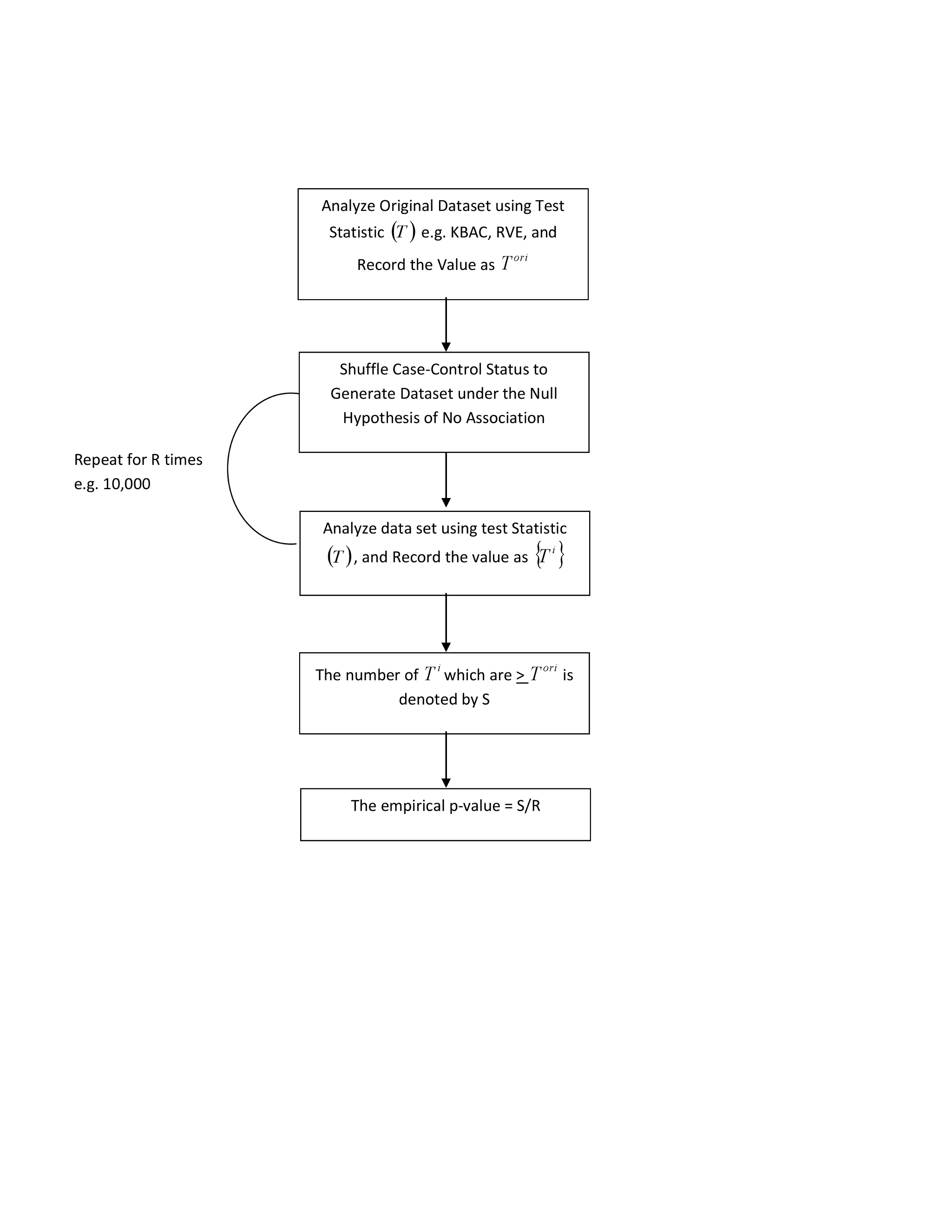

Supplement: Figure S1 — Schematic illustration of the permutation procedure used for evaluating statistical significance empirically. (0.19 MB TIF) [file pgen.1001156.s001.tif]

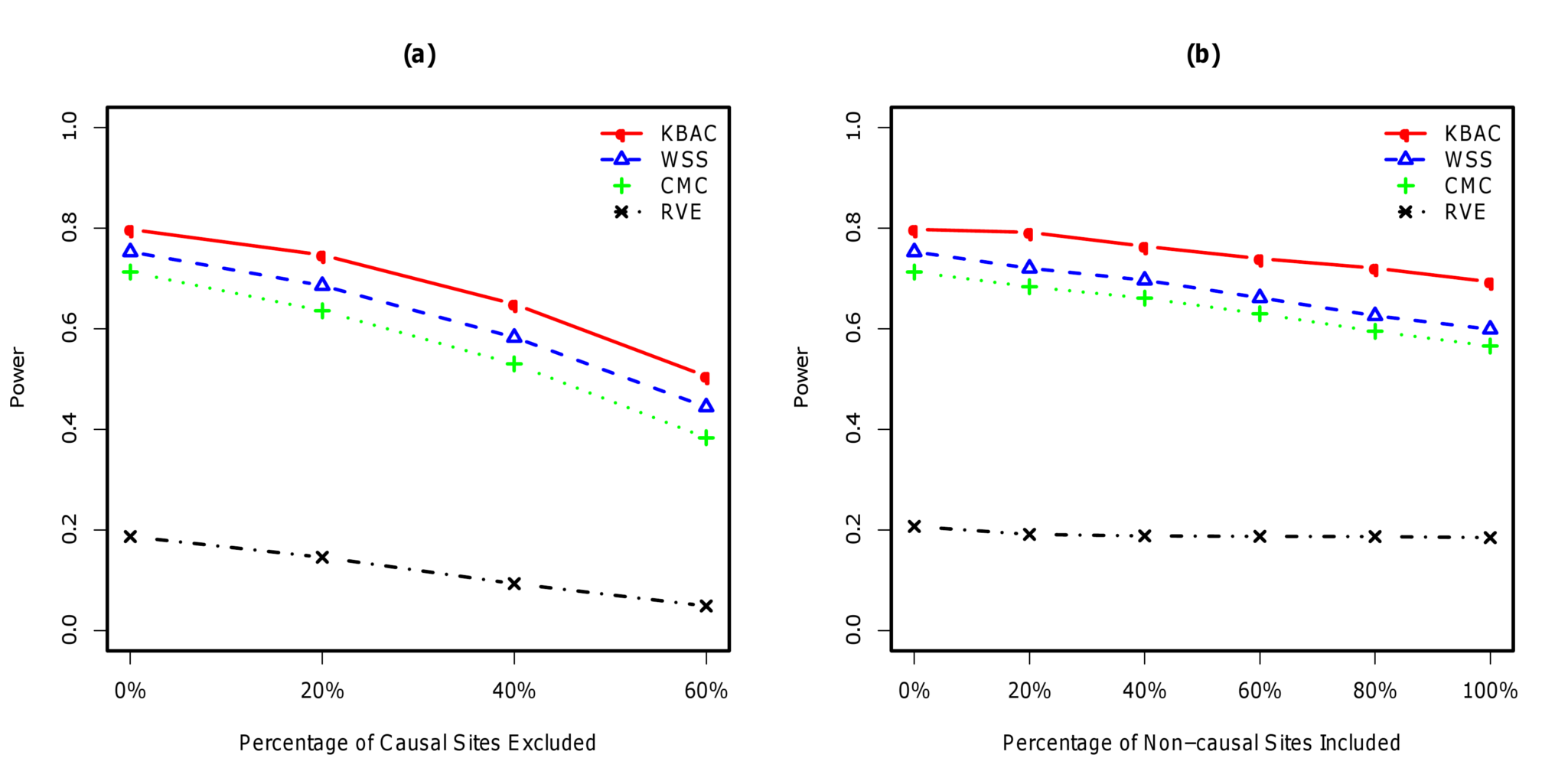

Supplement: Figure S2 — Impact of misclassifications under main effects model with fixed genetic effects using simulated SFS for EA. (0.18 MB TIF) [file pgen.1001156.s002.tif]

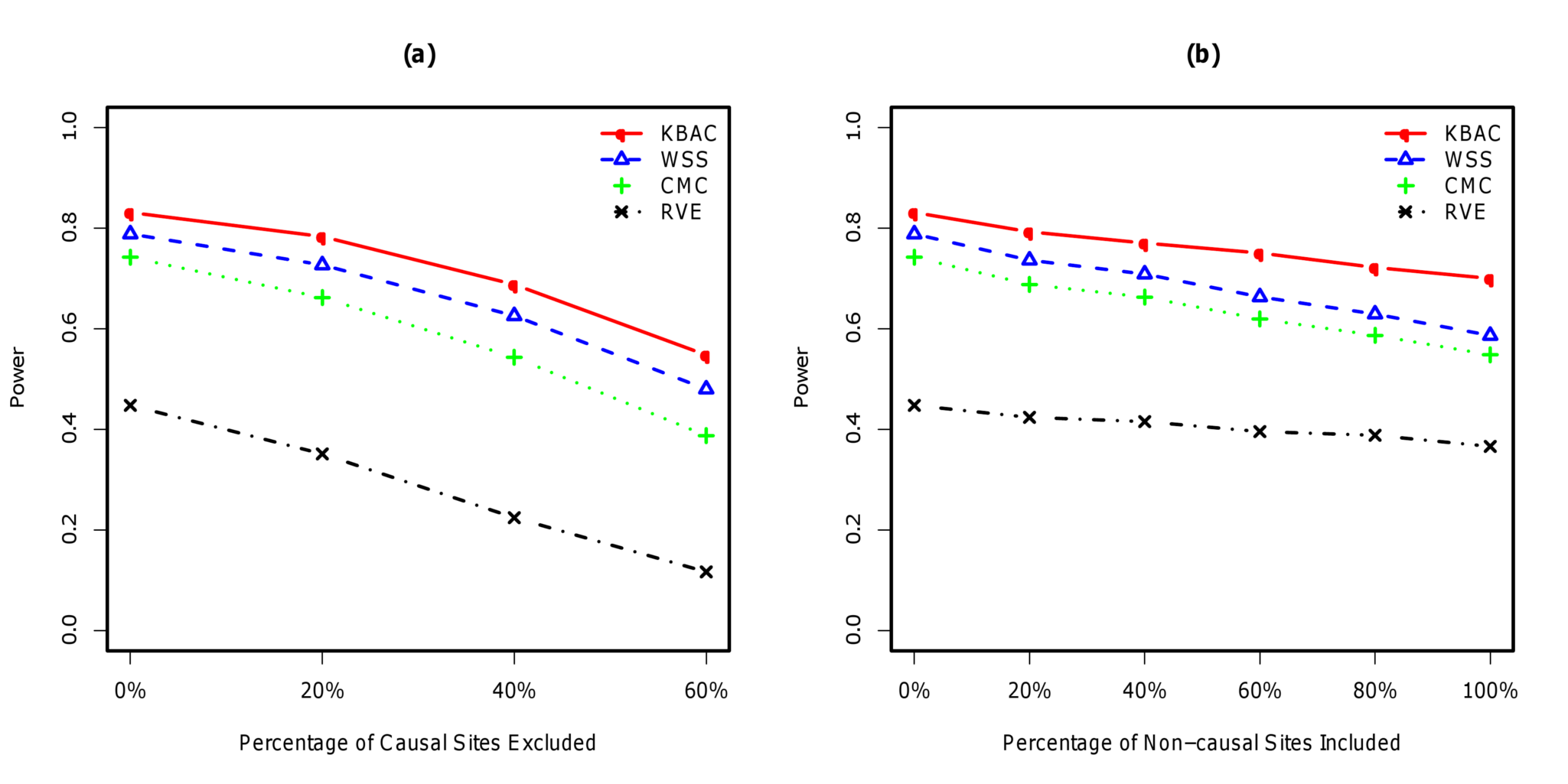

Supplement: Figure S3 — Impact of misclassifications under main effects model with variable genetic effects using simulated SFS for EA. (0.18 MB TIF) [file pgen.1001156.s003.tif]

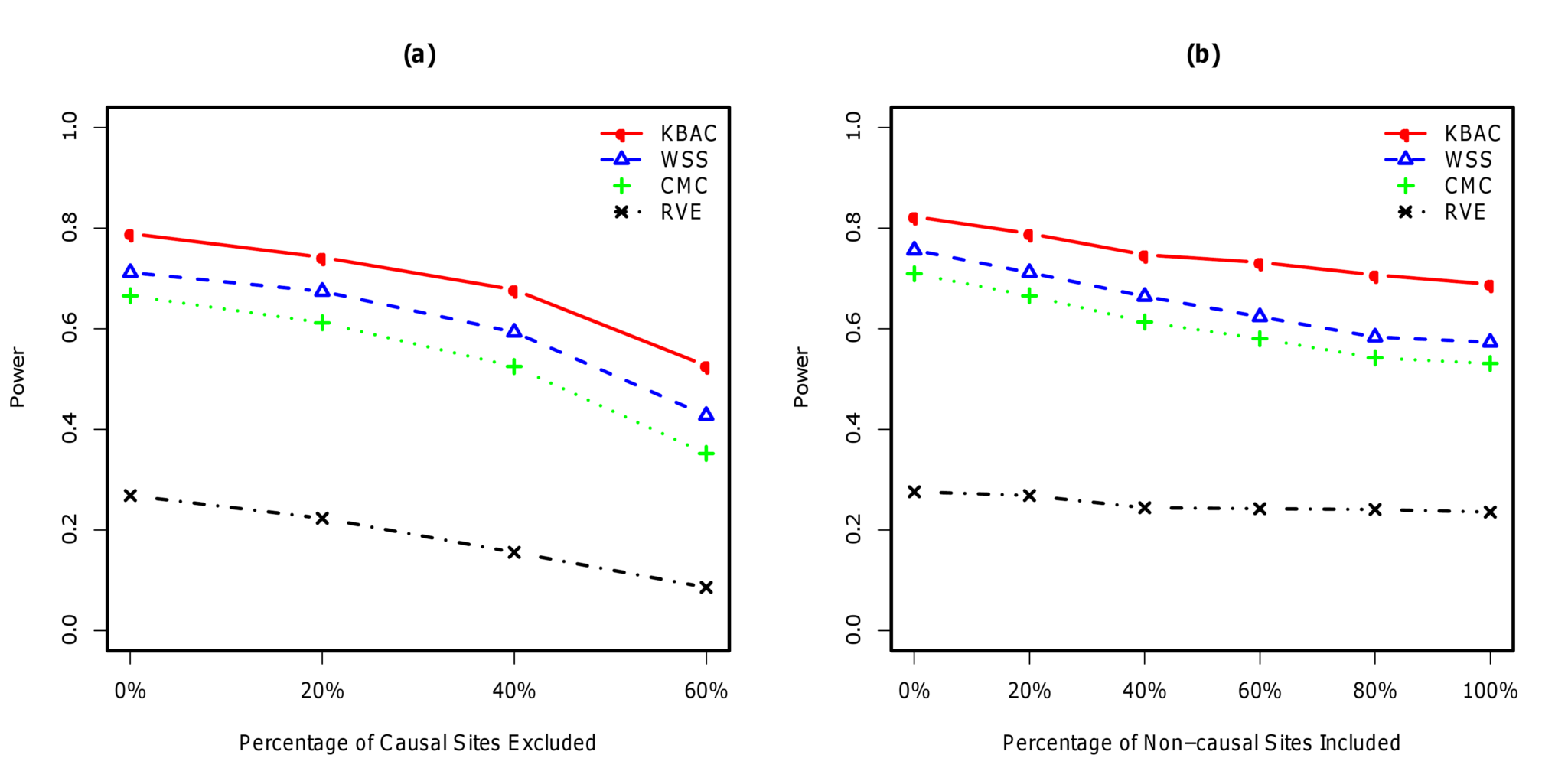

Supplement: Figure S4 — Impact of misclassifications under main effects model with fixed genetic effects using estimated SFS for AA from genes in ANGPTL family. (0.18 MB TIF) [file pgen.1001156.s004.tif]

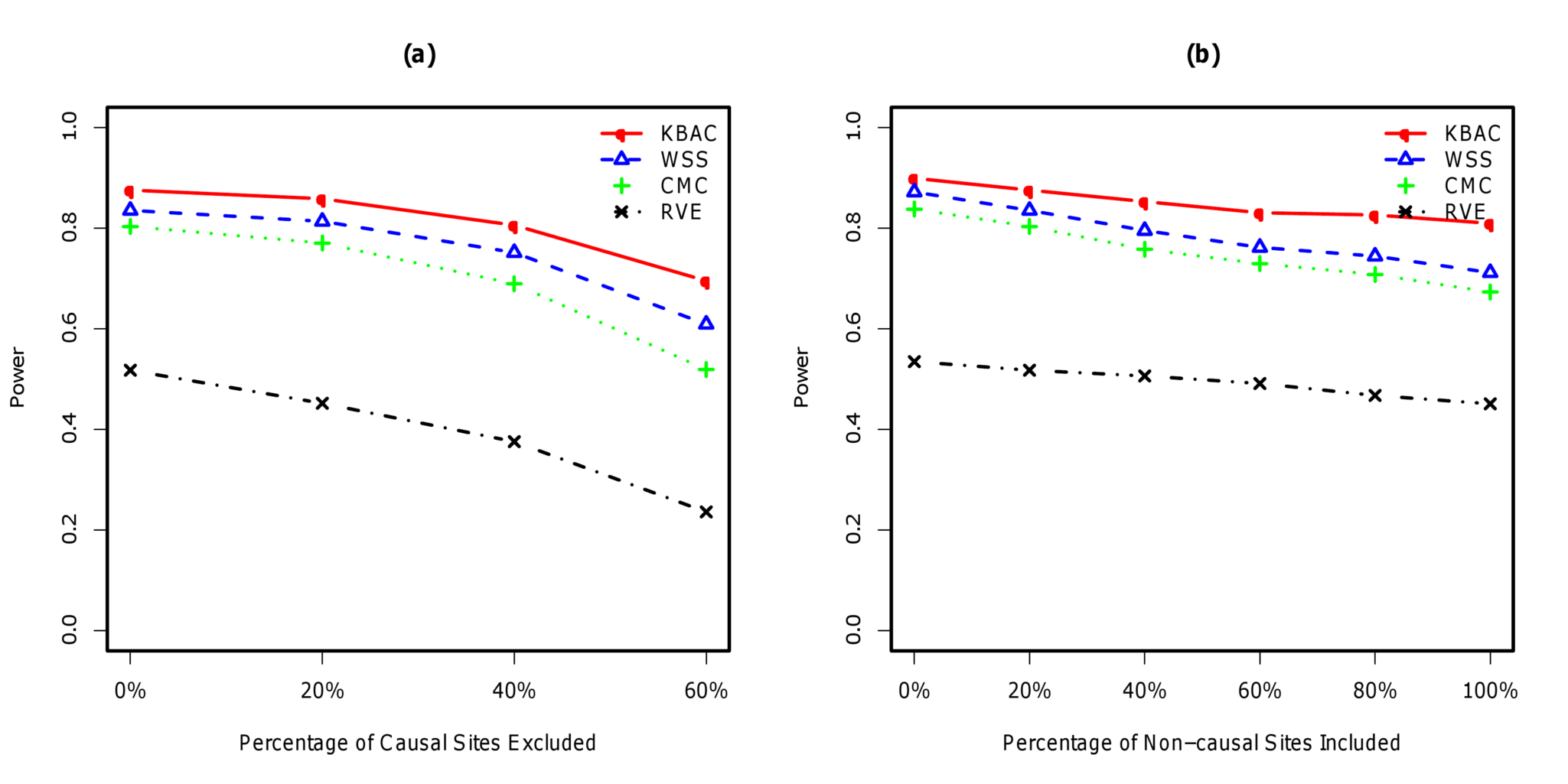

Supplement: Figure S5 — Impact of misclassifications under main effects model with variable genetic effects using estimated SFS for AA from genes in ANGPTL family. (0.18 MB TIF) [file pgen.1001156.s005.tif]

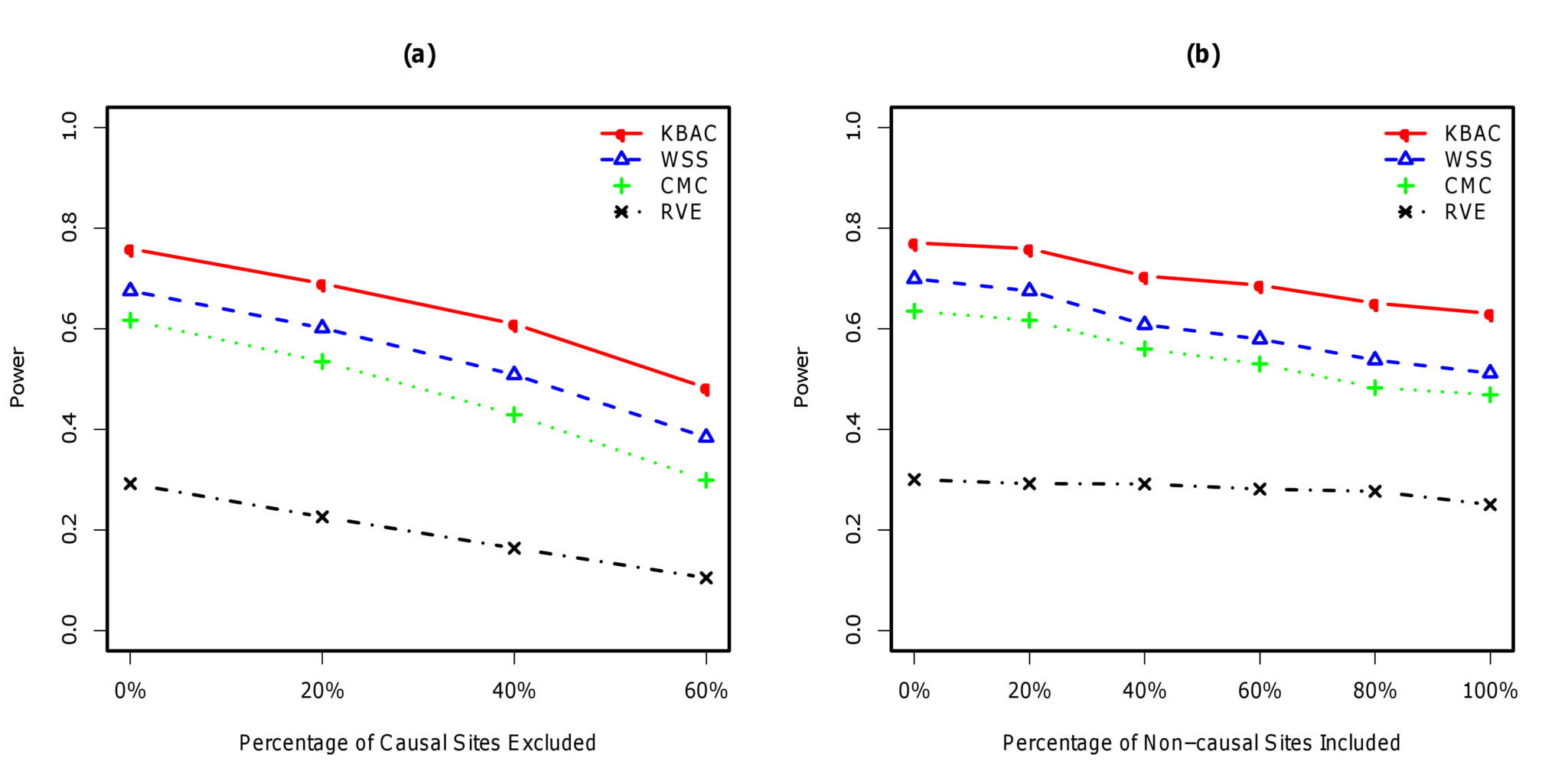

Supplement: Figure S6 — Impact of misclassifications under main effects model with fixed genetic effects using estimated SFS for EA from genes in ANGPTL family. (0.18 MB TIF) [file pgen.1001156.s006.tif]

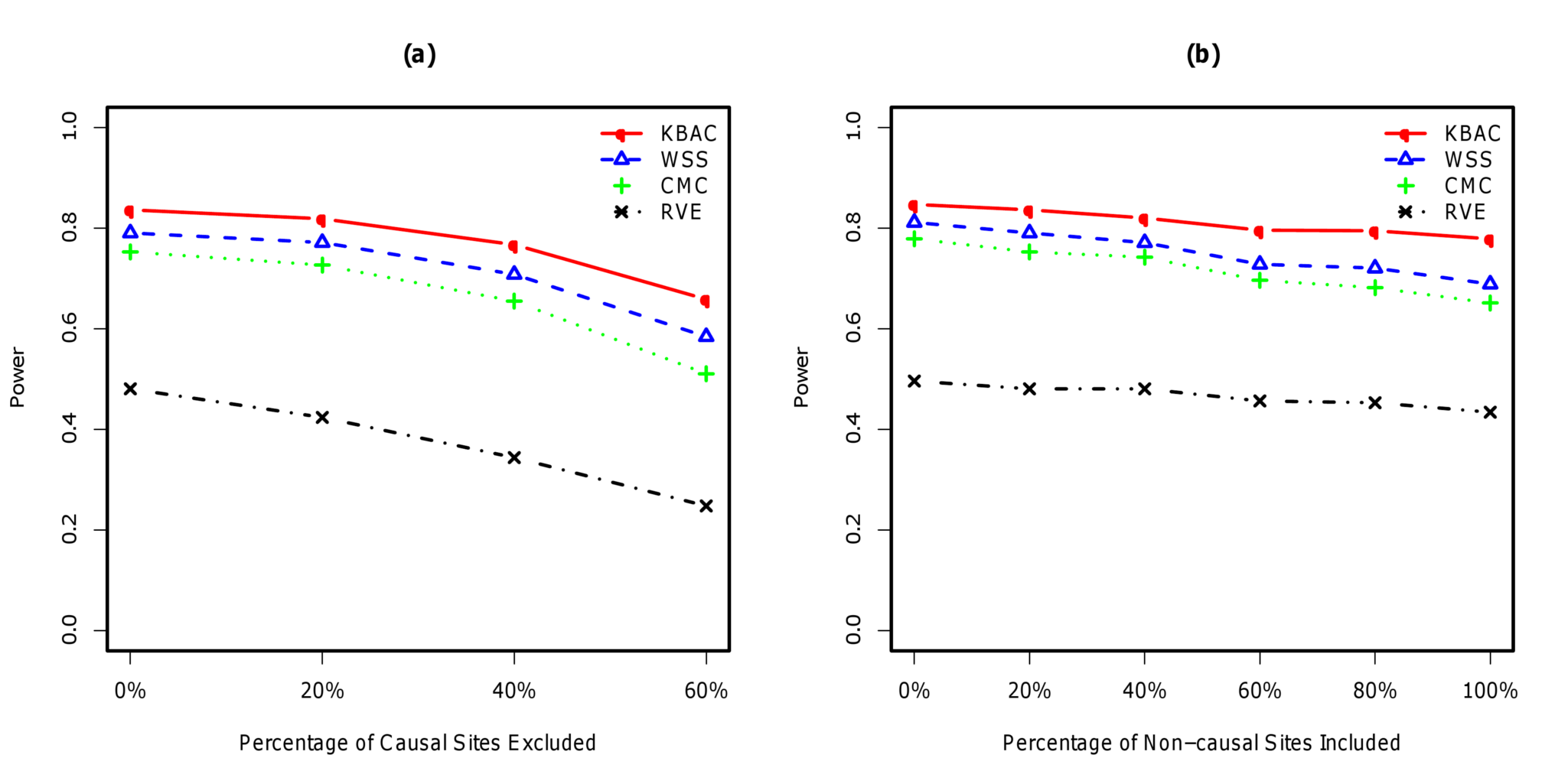

Supplement: Figure S7 — Impact of misclassifications under main effects model with variable genetic effects using estimated SFS for EA from genes in ANGPTL family. (0.18 MB TIF) [file pgen.1001156.s007.tif]

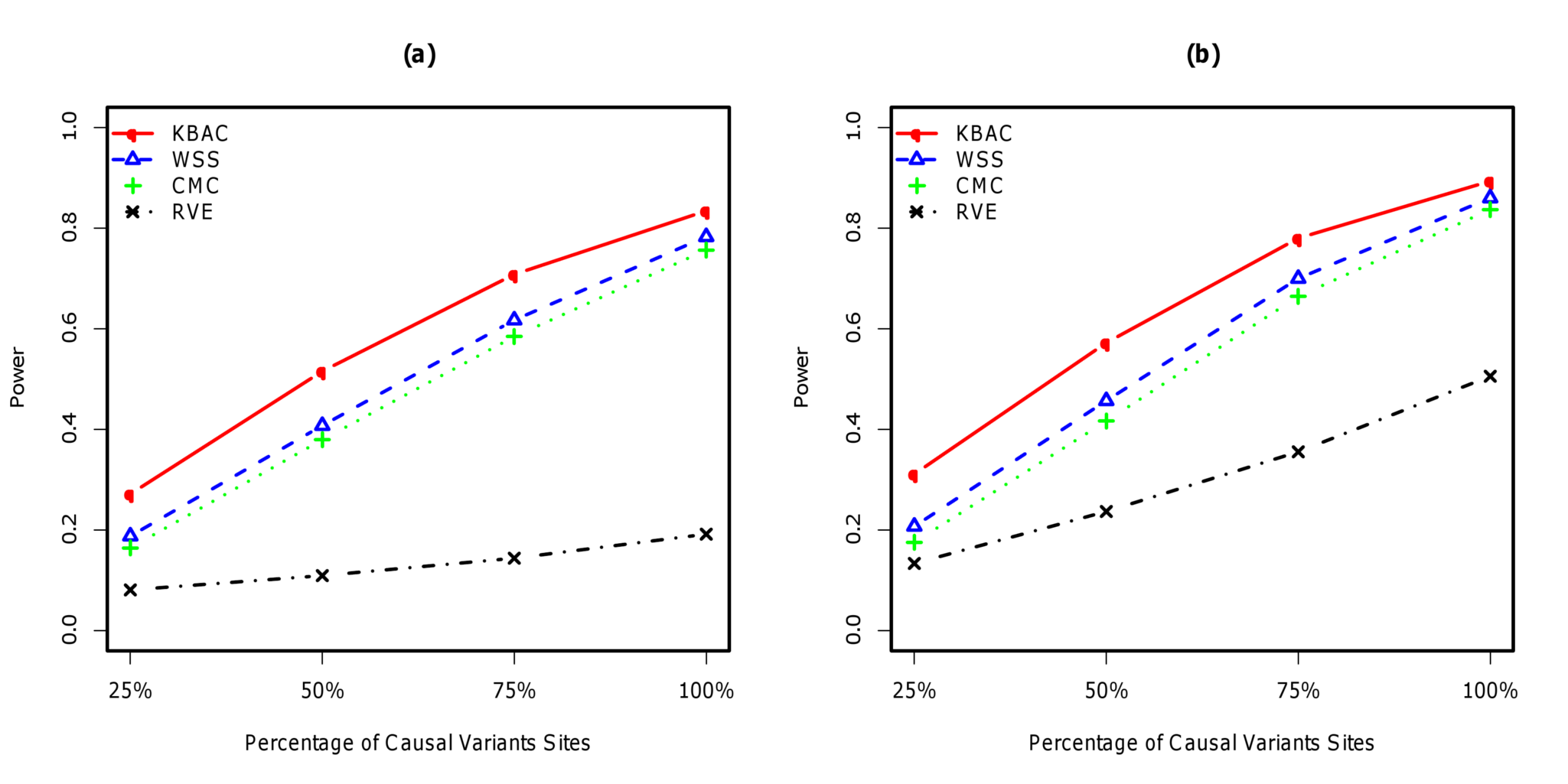

Supplement: Figure S8 — Power comparisons for within gene (left panel) and between gene interaction model (right panel) with simulated SFS for EA. (0.19 MB TIF) [file pgen.1001156.s008.tif]

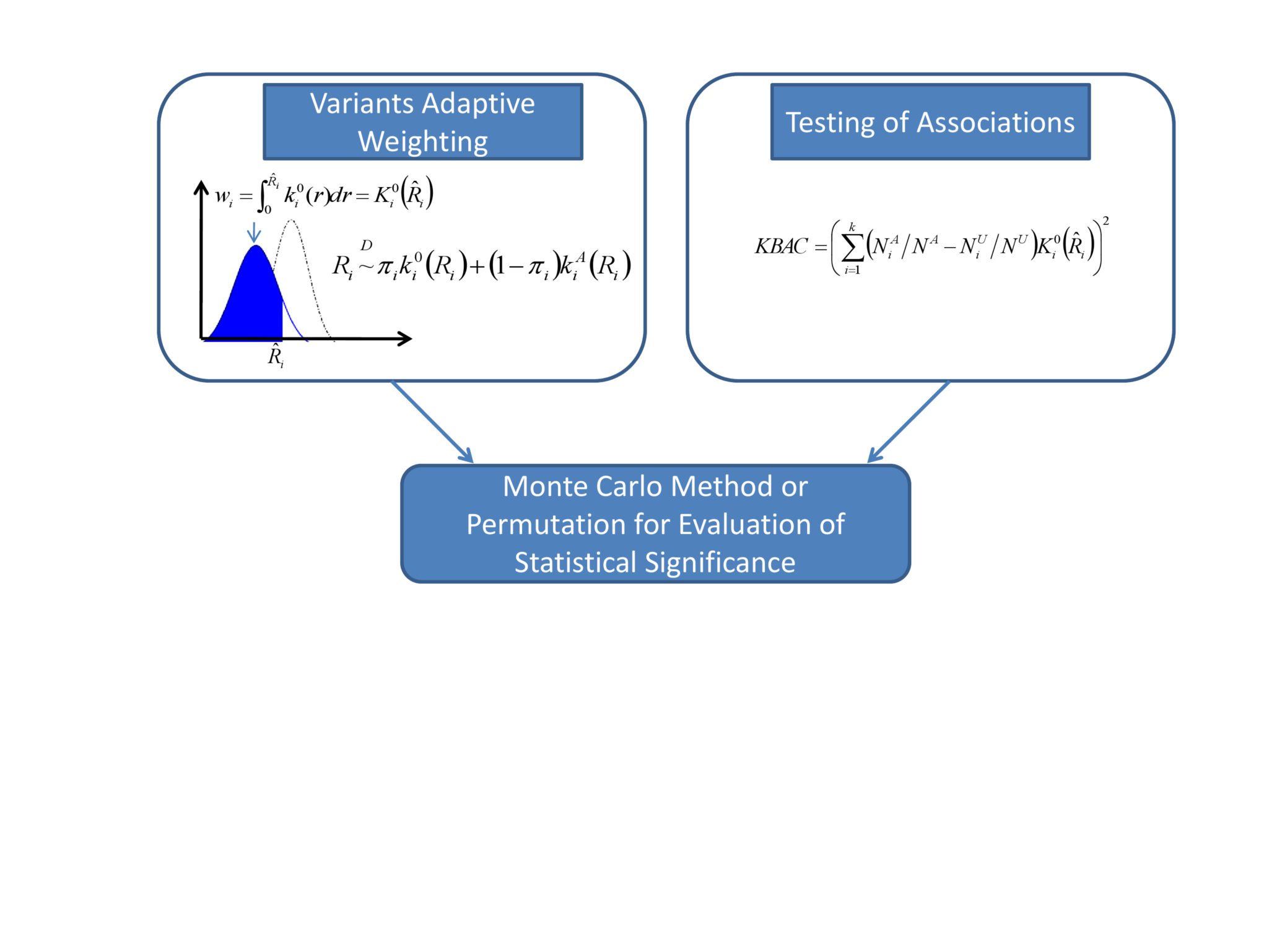

Supplement: Figure S9 — Graphical Illustrations for KBAC Statistic. In the KBAC framework, variants adaptive weighting and testing of associations are simultaneously performed. The statistical significance can be evaluated using either permutations or Monte Carlo approximations. For information on nomenclature used please refer to the Materials and Methods section. (0.25 MB TIF) [file pgen.1001156.s009.tif]

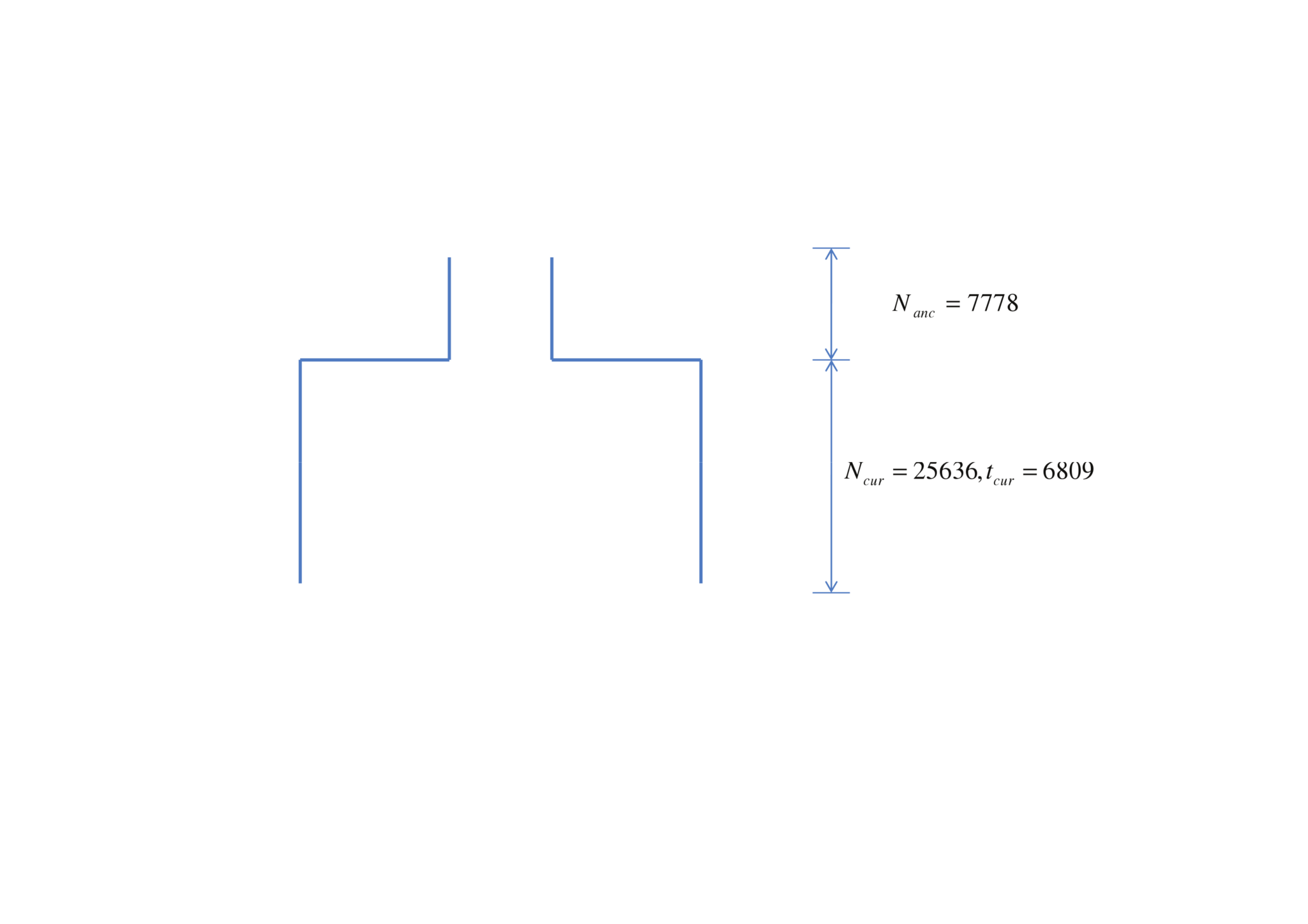

Supplement: Figure S10 — Demographic History of AA with Two-Epoch Change. (0.06 MB TIF) [file pgen.1001156.s010.tif]

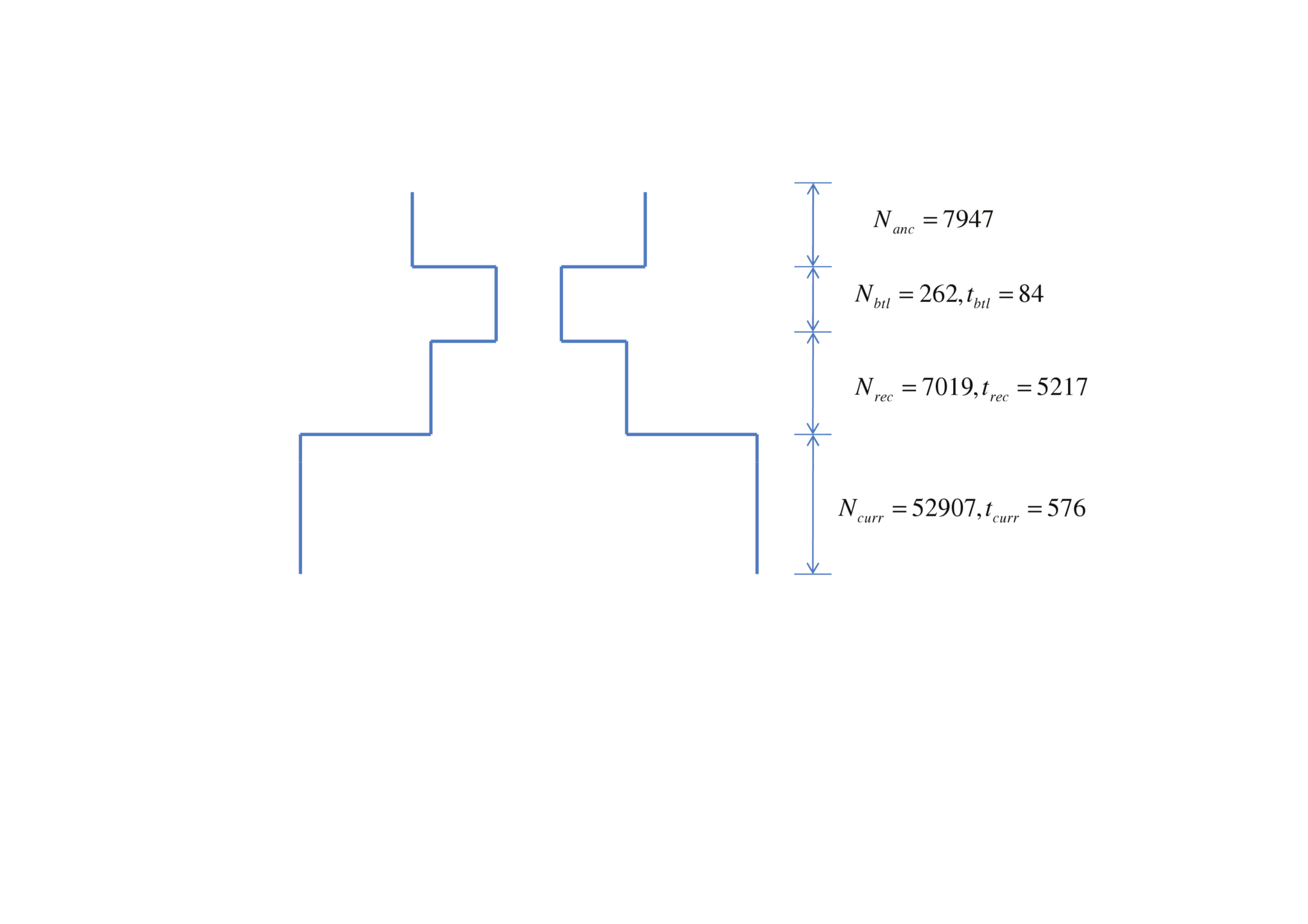

Supplement: Figure S11 — Complex Demographic History of EA. (0.09 MB TIF) [file pgen.1001156.s011.tif]
