# Supplementary material for: A Novel Adaptive Method for the Analysis of Next-Generation Sequencing Data to Detect Complex Trait Associations with Rare Variants Due to Gene Main Effects and Interactions
Source: PLoS Genet. 2010 Oct 14;6(10):e1001156. doi: 10.1371/journal.pgen.1001156 (PMC2954824; doi:10.1371/journal.pgen.1001156)
Supplement: Table S4 — Rare variant summary statistics. The summary statistics are displayed for the generated replicates under main effects model with fixed and variable genetic effects. Estimated SFS from EA population with ANGPTL dataset was used. Scenarios with different proportions of causal variants excluded and scenarios with different proportions of non-causal variants included were considered. The table displays for a given sample, the information on a) the average proportion of rare NS variant carriers among cases and controls; b) the mean number of rare NS variant sites; c) the mean number of rare NS variant sites that are exclusive to cases or controls; d) the average proportion of case and control rare NS variant carriers with more than one rare variant. For each scenario, a sample size of 1,000 cases and 1,000 controls were used. 2,000 replicates were generated for each scenario. (0.04 MB DOC) [file pgen.1001156.s015.doc]

| Scenario | | Rare Variant Carrier Frequencies in Cases/Controls | Mean Number of Rare Variant Sites | Mean number of Rare Variant Sites Observed Exclusively in Cases/Controls | Proportions of Rare Variant Carriers with More than One Rare Variant in Case/Controls |
| --- | --- | --- | --- | --- | --- |
| Phenotypic Model with Variable Genetic Effects Inversely Correlated with MAFs | | | | | |
| Percentage of Causal Variants Excluded | 20% | 0.027/0.009 | 4.43 | 1.203 | 0.011/0.004 |
|  | 40% | 0.021/0.007 | 3.478 | 0.955 | 0.008/0.002 |
|  | 60% | 0.015/0.005 | 2.489 | 0.686 | 0.005/0.002 |
| Percentage of Non-causal Variants Included | 0% | 0.032/0.011 | 5.446 | 1.472 | 0.013/0.004 |
|  | 20% | 0.035/0.013 | 6.146 | 1.744 | 0.015/0.005 |
|  | 40% | 0.037/0.015 | 7.335 | 2.214 | 0.016/0.006 |
|  | 60% | 0.039/0.018 | 8.256 | 2.499 | 0.017/0.008 |
|  | 80% | 0.042/0.02 | 9.43 | 2.993 | 0.019/0.01 |
|  | 100% | 0.044/0.022 | 10.514 | 3.376 | 0.02/0.01 |
| Phenotypic Model with Fixed Genetic Effects Unrelated to MAFs | | | | | |
| Percentage of Causal Variants Excluded | 20% | 0.022/0.009 | 4.424 | 1.228 | 0.009/0.004 |
|  | 40% | 0.017/0.007 | 3.472 | 0.981 | 0.007/0.002 |
|  | 60% | 0.012/0.005 | 2.474 | 0.692 | 0.004/0.002 |
| Percentage of Non-causal Variants Included | 0% | 0.027/0.011 | 5.414 | 1.543 | 0.012/0.004 |
|  | 20% | 0.029/0.013 | 6.124 | 1.753 | 0.012/0.006 |
|  | 40% | 0.032/0.016 | 7.347 | 2.257 | 0.014/0.006 |
|  | 60% | 0.034/0.018 | 8.241 | 2.539 | 0.015/0.008 |
|  | 80% | 0.036/0.02 | 9.234 | 2.937 | 0.016/0.009 |
|  | 100% | 0.039/0.022 | 10.504 | 3.439 | 0.018/0.01 |
