# Supplementary material for: A Novel Adaptive Method for the Analysis of Next-Generation Sequencing Data to Detect Complex Trait Associations with Rare Variants Due to Gene Main Effects and Interactions
Source: PLoS Genet. 2010 Oct 14;6(10):e1001156. doi: 10.1371/journal.pgen.1001156 (PMC2954824; doi:10.1371/journal.pgen.1001156)
Supplement: Text S1 — Supplementary Material. (0.25 MB DOC) [file pgen.1001156.s016.doc]

**A novel adaptive method for the analysis of next generation sequencing data to detect complex trait associations with rare variants due to gene main effects and interactions**

**Supplementary Material:**

Dajiang J. Liu & Suzanne M. Leal*

*: To whom the correspondence should be addressed

***Simulation of Demographic Model and Selections:***

According to parameters estimated in Boyko et al^1^, population genetic model incorporating demographic change and purifying selections were used to simulate African American (AA) and European American (EA) variant data. For AA, a simple two-epoch model with two degrees of freedom was used, where the population was constant with size $N_{anc}=7,778$ followed by an instant population expansion 6809 generation ago to its current size $N_{curr}=25,636$. It has been shown that this simple demographic model provides good fit to neutral rare variant frequency spectrums.

For EA, a more complicated demographic model with six degrees of freedom was needed to provide a marginal fit to neutral variant frequency spectrum. According to the model, the population was originally constant with size $N_{anc}=7,947$. A sharp bottle neck occurred with population size shrinking to $N_{btl}=262$. The bottleneck lasted 84 generations. The first instant population recovery immediately followed, during which the population size changed to $N_{rec}=7,019$. The population was constant in size afterwards for $t_{rec}=5,217$.The second population expansion took place 576 generation ago where the effective population size was changed to $N_{curr}=52,907$. Graphical illustrations of the demographic changes for AA and EA can be found in (**Supplemental Fig. 9 and 10**).

Selection was modeled as Gamma distribution, which has been shown to provide good fit to data, and at the same time being parsimonious^1^. The selective disadvantage of new heterozygous and homozygous mutations is assumed to be $s$ and $2s$. The distributions for fitness effects were estimated for scaled selective disadvantage $\gamma=2N_{curr}s$. For EA, the scaled selective disadvantage satisfies,

$$\gamma_{EA}=-x,x\sim\frac{\beta_{EA}^{\alpha_{EA}}}{\Gamma\left(\alpha_{EA}\right)}x^{\alpha_{EA}-1}\exp\left(-\beta_{EA}x\right)$$

where the parameters satisfy $\alpha_{EA}=0.206,\beta_{EA}=15,400$. For AA, the scaled selective disadvantage follows

$$\gamma_{AA}=-x,x\sim\frac{\beta_{AA}^{\alpha_{AA}}}{\Gamma\left(\alpha_{AA}\right)}x^{\alpha_{AA}-1}\exp\left(-\beta_{AA}x\right)$$

and the parameters follow $\alpha_{AA}=0.184,\beta_{AA}=8,200$.

***Estimation of Rare Variant Frequency Spectrums:***

The estimation of demographic model and selections simultaneously are challenging^2^, as a result, in order to further evaluate the performance of KBAC against other proposed statistical methods, we use rare variant frequency spectrums computed from large re-sequencing dataset with simulated phenotype. One data set that is suitable for this purpose is the *ANGPTL* dataset from Dallas Heart Study^3^. The data set is one of the largest existing population re-sequencing data set, where population samples composed of three different ethnic groups (EA, AA, and Hispanic Americans (HA)) were ascertained and sequenced for *ANGPTL* 3, 4, 5 and 6. Site frequency spectrums (SFS) for EA and AA were estimated. The sample size for HA is too small to estimate rare variant frequency spectrums.

When estimating rare variant minor allele frequencies (MAF) , random mating is assumed within each ethnic population. For a sample of size $N$, the number of rare variants observed at nucleotide site $j$ follows a truncated binomial distribution, i.e.

$$P\left(K_{j}=k;2N,p_{j}\right)=\frac{\left(\begin{matrix} 2N \\ k \end{matrix}\right)p_{j}^{k}\left(1-p_{j}\right)^{2N-k}}{1-\left(1-p_{j}\right)^{2N}}$$

A method of moment (MOM) estimator for MAF $p_{j}$can be constructed using the first moment from the above truncated binomial distribution, i.e.

$$E\left(K_{j}\right)=\frac{2Np_{j}}{1-\left(1-p_{j}\right)^{2N}}$$

Our experience indicates that the MOM estimator performs consistently better than naïve estimators for low frequency variants.

***Mathematical Descriptions of Genetic Models:***

***Gene Main Effects Model with Constant Genetic Effects:***

The M-sites genotype is denoted by $G=\left(g_{1},\cdots,g_{d},g_{d+1},\cdots,g_{M}\right),$ where alleles in sites 1 through $d$affect the disease phenotype of interests while sites $d+1$ through $M$ do not. The genotype $G$ is coded such that $g_{j}$ is the number of rare variants observed at site, so $g_{j}=2$ if site is homozygous for the rare alleles, $g_{j}=1$ if site $j$ is heterozygous, and $g_{j}=0$ if site is homozygous for the common allele. Disease odds ratios are determined by multi-site genotype at sites 1 through $d$. Each mutation at sites 1 through $d$ increases the disease odds by 3 folds and the model can be represented by the following equation:

$\log\left(\frac{P\left(A|G\right)}{1-P\left(A|G\right)}\right)=\beta_{0}+\beta_{1}\times\left(\sum_{j=1}^{d} g_{j}\right)$ (S1).

If the penetrance for wild-type genotype is set to be 0.01, the parameters $\beta_{0},\beta_{1}$ are accordingly given by

$\beta_{0}=\log\left(\frac{0.01}{1-0.01}\right),\beta_{1}=\log\left(3\right)$ (S2).

In the examination of the impact of variant misclassification, different proportions of disease causal variants (variants in sites 1 through $d$) and non-causal variants (variants in sites $d+1$to$M$) are included or excluded from the analyses.

***Gene Main Effects Model with Genetic Effects Inversely Correlated with MAF***

Similar setups are used as main effects model with fixed genetic effects. The M-sites genotype is denoted by$G=\left(g_{1},\cdots,g_{d},g_{d+1},\cdots,g_{M}\right),$, where alleles in sites 1 through $d$ affect the disease phenotype of interests while sites $d+1$ through $M$do not. The MAF for causal variants are given by $p_{1},p_{2},\cdots,p_{d}$. The maximum and minimum of the causal variants frequencies were denoted by $p_{\max},\text{ }p_{\min}$ respectively, i.e.

$$p_{\max}=\max\left\{p_{1},p_{2},\cdots,p_{d}\right\},p_{\min}=\min\left\{p_{1},p_{2},\cdots,p_{d}\right\}$$

The disease odds $\exp\left(\beta_{1j}\right)$ for variant $j$ is determined by linear interpolation between $OR_{\max}$ and $OR_{\min}$, i.e.

$$\beta_{1j}=\log\left(OR_{\max}\right)-\frac{p_{j}-p_{\min}}{p_{\max}-p_{\min}}\times\left(\log\left(OR_{\max}\right)-\log\left(OR_{\min}\right)\right)$$

As a result, causal variants with the lowest frequency have the highest disease odds, while those with the highest frequency have the smallest disease odds. The causal variants’ disease odds satisfy an exponential functional relation with their MAFs. The model shares similar properties with the model introduced in Madsen and Browning^4^, i.e. the disease odds ratio is negatively correlated with the allele frequencies. However, our model is more realistic for complex phenotypes, where the genetic effects of rare variants are bounded and are not large enough to cause familial aggregations^5^. We set $OR_{\max}=20,\text{ }OR_{\min}=2$. As a large proportion of rare variants have very low frequencies and may not be uncovered in disease studies, with the choice of $OR_{\max},\text{ }OR_{\min}$, a majority of causal variants uncovered in the sample will have ORs between 2 to 4. This is compatible with the surveys of multi-factorial diseases^5^.

The probability of being affected is determined by multi-site genotype at sites through . Log odds ratio of causal rare variants at site $j$ is given by $\beta_{1j}$ and the model can be represented by the following equation:

$\log\left(\frac{P\left(A|G\right)}{1-P\left(A|G\right)}\right)=\beta_{0}+\left(\sum_{j=1}^{d} \beta_{1j}g_{j}\right)$ (S1).

If the penetrance for wild-type genotype is set to be 0.01, the intercept parameter $\beta_{0}$ is accordingly given by

$\beta_{0}=\log\left(\frac{0.01}{1-0.01}\right)$ (S2).

In the examination of the impact of variant misclassification, different proportions of disease causal variants (variants in sites 1 through $d$) and non-causal variants (variants in sites $d+1$ to$M$) are included or excluded from the analyses.

***Gene Interaction Model:***

***Within gene interaction model:***

The within gene interaction model, where a common variant in the promoter region and multiple rare variants in the exonic regions interact, is motivated by Hirschsprung’s disease and the hypothesized interaction within the *RET* gene. The genetic effects of rare coding variants are hypothesized to be modulated by a common variant in the promoter region in a dosage dependent fashion^6,7^. As a *cis-trans* effect is suggested^6,7^, haplotype is introduced in this model. Two $M+1$-site haplotypes are represented by $H_{1}=\left(h_{10},h_{11},\cdots,h_{1d},h_{1,d+1}\cdots,h_{1M}\right)$,$H_{2}=\left(h_{20},h_{21},\cdots h_{2d},h_{2,d+1}\cdots,h_{2M}\right)$, where site 0 is polymorphic and sites 1 through $M$are rare variant sites. Rare variants in sites 1 through $d$ are causal, and rare variants in sites $d+1$ through $M$ are non-causal. The haplotypes are coded such that each entry is the number of minor alleles observed at the corresponding site. The rare causal variants will increase the disease odds ratio by 3 folds, only if they lie on the same haplotype as the minor allele in site 0. Those lying on the haplotype with the major allele at site 0 do not elevate disease odds. The log odds for each rare variant are additive. Quantitatively, the disease model can be expressed as

$\log\left(\frac{P\left(A|H_{1},H_{2}\right)}{1-P\left(A|H_{1},H_{2}\right)}\right)=\beta_{0}+\beta_{1}\times\left[\delta\left(h_{10}=1\right)\left(\sum_{j=1}^{d} h_{1j}\right)+\delta\left(h_{20}=1\right)\left(\sum_{j=1}^{d} h_{2j}\right)\right]$(S3).

Similar to the gene main effects model, the parameters $\beta_{0}$ and $\beta_{1}$are given by

$\beta_{0}=\log\left(\frac{0.01}{1-0.01}\right),\text{ }\beta_{1}=\log\left(3\right)$(S4).

Scenarios with different proportions of causal variants i.e. 25% to 100% were considered in the simulation studies to examine the impact of misclassification.

***Interaction model between rare variants in different genes:***

The example of interaction between rare variants in two different genes is based upon observations in breast cancer where rare variants in the CHEK2 gene increase disease risk when BRCA gene is wild type, and BRCA gene will elevate disease risk regardless of the genotype of CHEK2^8^. Two multiple site genotypes are denoted as$G_{1}=\left(g_{11},\cdots,g_{1d_{1}},g_{1,d_{1}+1}\cdots,g_{1M_{1}}\right),\text{ }G_{2}=\left(g_{21},\cdots,g_{2d_{2}},g_{2,d_{2}+1},\cdots,g_{2M_{2}}\right)$. The causal rare variants in gene 2 will each increase disease odds two-fold when there are no causal rare variants in gene 1, and high risk causal variants in gene 1 will each increase disease odds four-fold regardless of the multi-site genotype of gene 2. Mathematically, the disease model is given by

$\log\left(\frac{P\left(A|G_{1},G_{2}\right)}{1-P\left(A|G_{1},G_{2}\right)}\right)=\beta_{0}+\beta_{1}\times\left[\sum_{j=1}^{d_{1}} g_{1j}\right]+\beta_{2}\times\delta\left[\left(\sum_{j=1}^{d_{1}} g_{1j}\right)=0\right]\times\left(\sum_{j=1}^{d_{2}} g_{2j}\right)$(S5)

By matching model coefficients with genetic parameters,

$\beta_{0}=\log\left(\frac{0.01}{1-0.01}\right),\beta_{1}=\log\left(4\right),\beta_{2}=\log\left(2\right)$(S6),

are obtained.

Similar to within gene interaction model, scenarios with different proportions of causal variants, i.e. 25% ~ and 100%, were considered in the simulation studies to examine the impact of misclassifications.

***Assessment of the Impact of Phenotypic model and SFS on Power Comparisons:***

Additional simulation studies were conducted to evaluate the impact of different phenotypic models and the influence of different choices of SFS on the power comparisons between KBAC, WSS, CMC and RVE. Specifically,

1. simulated SFS for EA population were used with two types of main effects phenotypic models (**Supplemental Fig. 2 and 3 , Supplemental Table 1**) and two interaction models (**Supplemental Fig. 8, Supplemental Table 2**).
2. estimated SFS for AA population from ANGPTL dataset were used with two types of main effects phenotypic models (**Supplemental Fig. 4 and 5, Supplemental Table 3**)
3. estimated SFS for EA population were used with two types of main effects phenotypic models (**Supplemental Fig. 6 and 7**)

As estimated SFS from only four genes is available, interaction models were not investigated for estimated SFS.

Very similar results are observed for simulations using simulated SFS as well as estimated SFS. Several results are discussed below (particularly on different choices of SFS):

1. For simulations using estimated SFS, higher diversity was observed for replicates simulated based on AA population than EA population. For example, when all the variants were included using the model with fixed genetic effects, for a sample of 1000 cases and 1000 controls, 11.2 sites for AA and 10.5 sites for EA were uncovered. One possible reason for this difference is that a larger sample size was collected for AA population in DHS than EA population. As a result, a higher fraction of nucleotide sites (and a higher total frequency of rare variants) were uncovered for the AA population. Another possible reason is that populations with African ancestry tend to have longer population history, larger effective population size and exhibit higher genetic diversity.
2. Power comparisons under different demographic and phenotypic model are largely unchanged. The results presented are robust for different choices of alternative hypothesis.

**References**

1. Boyko, A.R. et al. Assessing the evolutionary impact of amino acid mutations in the human genome. *PLoS Genet* **4**, e1000083 (2008).

2. Nielsen, R., Hellmann, I., Hubisz, M., Bustamante, C. & Clark, A.G. Recent and ongoing selection in the human genome. *Nat Rev Genet* **8**, 857-68 (2007).

3. Victor, R.G. et al. The Dallas Heart Study: a population-based probability sample for the multidisciplinary study of ethnic differences in cardiovascular health. *Am J Cardiol* **93**, 1473-80 (2004).

4. Madsen, B.E. & Browning, S.R. A groupwise association test for rare mutations using a weighted sum statistic. *PLoS Genet* **5**, e1000384 (2009).

5. Bodmer, W. & Bonilla, C. Common and rare variants in multifactorial susceptibility to common diseases. *Nat Genet* **40**, 695-701 (2008).

6. Fitze, G. et al. Functional haplotypes of the RET proto-oncogene promoter are associated with Hirschsprung disease (HSCR). *Hum Mol Genet* **12**, 3207-14 (2003).

7. Fitze, G. et al. Novel intronic polymorphisms in the RET proto-oncogene and their association with Hirschsprung disease. *Hum Mutat* **22**, 177 (2003).

8. Stratton, M.R. & Rahman, N. The emerging landscape of breast cancer susceptibility. *Nat Genet* **40**, 17-22 (2008).
